# Spatiotemporal monitoring of the rare northern dragonhead (*Dracocephalum ruyschiana*, Lamiaceae) — SNP genotyping and environmental niche modeling herbarium specimens

**DOI:** 10.1002/ece3.9187

**Published:** 2022-08-12

**Authors:** Malene Nygaard, Alexander Kopatz, James M. D. Speed, Michael D. Martin, Tommy Prestø, Oddmund Kleven, Mika Bendiksby

**Affiliations:** ^1^ NTNU University Museum Norwegian University of Science and Technology Trondheim Norway; ^2^ Natural History Museum and Botanical Garden University of Agder Kristiansand Norway; ^3^ Norwegian Institute for Nature Research Trondheim Norway; ^4^ Natural History Museum University of Oslo Norway

**Keywords:** biodiversity conservation, environmental niche modelling, herbarium specimens, microfluidic SNP genotyping, spatiotemporal stasis

## Abstract

The species we have studied the spatiotemporal genetic change in the northern dragonhead, a plant species that has experienced a drastic population decline and habitat loss in Europe. We have added a temporal perspective to the monitoring of northern dragonhead in Norway by genotyping herbarium specimens up to 200 years old. We have also assessed whether northern dragonhead has achieved its potential distribution in Norway. To obtain the genotype data from 130 herbarium specimens collected from 1820 to 2008, mainly from Norway (83) but also beyond (47), we applied a microfluidic array consisting of 96 SNP markers. To assess temporal genetic change, we compared our new genotype data with existing data from modern samples. We used sample metadata and observational records to model the species' environmental niche and potential distribution in Norway. Our results show that the SNP array successfully genotyped all included herbarium specimens. Hence, with the appropriate design procedures, the SNP array technology appears highly promising for genotyping old herbarium specimens. The captured genetic diversity correlates negatively with distance from Norway. The historical‐modern comparisons reveal similar genetic structure and diversity across space and limited genetic change through time in Norway, providing no signs of any regional bottleneck (i.e., spatiotemporal stasis). The regional areas in Norway have remained genetically divergent, however, both from each other and more so from populations outside of Norway, rendering continued protection of the species in Norway relevant. The ENM results suggest that northern dragonhead has not fully achieved its potential distribution in Norway and corroborate that the species is anchored in warmer and drier habitats.

## Graphical abstract

Our study provides new insight to guide conservation priorities for the charismatic flowering plant called northern dragonhead (*Dracocephalum ruyschiana*). It also showcases a fruitful integration of methods and data sources that jointly enables a holistic species assessment covering space and time.

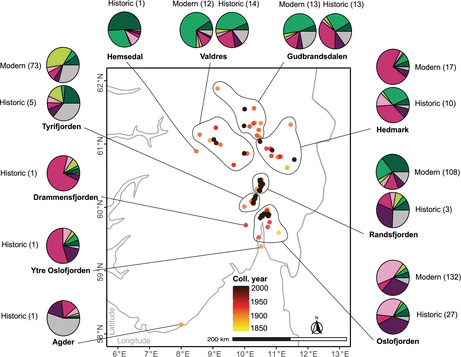

## 1 INTRODUCTION

Loss of biodiversity is one of the great challenges facing our society today. In order to predict how climatic and other changes will affect biodiversity, holistic knowledge is needed, from the level of genes to ecosystems. Drivers of biodiversity act both across space and through time. Holistic biodiversity studies should therefore include both spatial and temporal data of various kinds. Contemporary genetic, distributional, and ecological data provide a spatial snapshot across the latest generations only. In the absence of real‐time historical genetic data, various methods have been developed for approximating the past and predicting the future impacts of change (i.e., the temporal aspect) based on contemporary data alone. Liberating genetic, distributional, and ecological data from archived biological specimens, however, would enable to create a solid base to study temporal biodiversity dynamics in real time.

Archived biological collections, such as herbaria, fungaria, and seed, culture, in vitro, tissue, and DNA collections, contain expert‐curated specimens and associated information (i.e., metadata) collected throughout the world, some of which are several 100 years old. Such scientific collections provide verifiable records of the existence of an organism at a given time and place. With the exception of a few studies, such biological specimen archives have remained a largely untapped resource to study trajectories and trends of genetic diversity (Andrew et al., 2018; Bieker & Martin, 2018), one of the main reasons being recalcitrant DNA in old specimens. *Post‐mortem* degradation of DNA is an inherent trait and unending process of biological materials challenging the usability of archived biological specimens in DNA studies (Allentoft et al., 2012).

With new developments in genomic approaches, genetic data can now be liberated from historical specimens enabling their use as a source for genome‐scale biodiversity studies (museomics; Besnard et al., 2018). A second challenge is the lack of standardized, cost‐ and time‐efficient methods for capturing genomic data from historical DNA. Most of the genome‐scale approaches currently in use (e.g., shotgun deep sequencing, genome skimming, targeted DNA capture, and de novo organellar genome assembly; see Kistler et al., 2020, and references therein) are still both laborious and expensive and thus are, at this time, of less practical use in large‐scale species monitoring and biodiversity assessments. As biodiversity dynamics have no borders or fixed scales, informative biodiversity research calls for large‐scale assessments. Hence, there is a need for cheaper and more effective solutions for large‐scale biodiversity assessments.

Combining museomics (i.e., genomic analysis of museum specimens) with eco‐informatics (i.e., analyses of specimen occurrence data and ecological information) promises to be a fruitful integration of scientific domains, enabling more holistic species knowledge to guide monitoring efforts, in which knowledge about a species' behavior in relation to external forces over time is key. While genomic data can provide an evolutionary framework and delimit units at which selection is operating, georeferenced herbarium records provide basic occurrence data that can be used to understand, predict, and map species distributions and examine past phenological trends and even species interactions (e.g., Meineke et al., 2018). Occurrence data combined with biotic and abiotic data may further reveal key predictors of the species distributions by surveying various potentially explanatory variables. At times when specimen label information was available only from local, internal databases and often required assistance from collection staff, few researchers made the effort to collect such data. With recent global digitization initiatives, distributional and ecological specimen label information are rapidly becoming readily available through public repositories (e.g., GBIF.org, 2020). Such evolution‐ecology integration for species monitoring has been practised for some time (e.g., Bendiksby et al., 2014; Carlsen et al., 2012; Nygaard et al., 2021). Herbarium specimens were an essential data source in these studies, but the temporal dimension was not specifically addressed.

Plants are key components of biodiversity, contributing to ecosystem resilience and services that we depend upon. The world's 178 herbaria (archived collections of plants) contain about 390 million specimens collected throughout the world for more than 350 years (Thiers, 2020). In the present study, we add a historic level to the species monitoring of the flowering plant species, *Dracocephalum ruyschiana* L. (northern dragonhead; Lamiaceae) by testing a microfluidic‐based SNP genotyping array on old herbarium specimens. This approach has recently been applied by others to historical materials of, for example, tropical tree species and salmon (Finch et al., 2020; Johnston et al., 2013; Östergren et al., 2021).

Northern dragonhead is considered a remnant of the glacial steppe flora in Europe with its westernmost occurrences in France and Scandinavia (Lazarević et al., 2009). The typical northern dragonhead habitats throughout its distribution have been, and still are, subject to alteration and destruction by for instance succession (due to ceases of traditional agricultural use like grazing and mowing), increased feralization (due to intensification of farming), and development of infrastructure (due to e.g., use of herbicides along train rails; Norwegian Directorate for Nature Management, 2010; Økokrim, 2013). As future conflicts seem inevitable, it was decided that all conservation options should be assessed, including ex situ conservation (IUCN/SSC, 2014). Translocation is one such method, which has been successfully performed on an entire northern dragonhead population in Norway (Natural History Museum, 2010); the original population was divided into two new localities, of which one is in the Botanical Garden in Oslo, where also viable seeds are curated. The success of translocations depends on several factors, including the choice of suitable habitats (see Schäfer et al., 2020, and references therein). Gaining knowledge about species' ecological requirements may improve the success rate of translocations.

More than 25% of the total European population of northern dragonhead is found in Norway, where it is one of three vascular plants that have a separate percept of law with an action plan for conservation (Lovdata, 2011; Norwegian Directorate for Nature Management, 2010). Northern dragonhead is listed as vulnerable on the Norwegian Red List of 2021 with an estimated 20–40% of populations having vanished during the period 1975–2020 due to reduction in suitable habitats (Solstad et al., 2021). Although its distribution throughout Europe is also highly fragmented, the northern dragonhead was classified as Least Concern in the most recent version of the IUCN Red List of Threatened Species (Ericsson et al., 2011). In Norway, northern dragonhead occurs exclusively in the southeastern part of the country. The most common habitats in Norway are dry, calcareous meadows, calcareous rocky outcrops along roads and railways, and extensively managed agricultural lands (Fægri & Danielsen, 1996; Norwegian Directorate for Nature Management, 2010).

Northern dragonhead is a diploid (2*n* = 2*x* = 14), insect‐pollinated, perennial herb (Fægri & Danielsen, 1996; Kyrkjeeide et al., 2020). It grows from a rhizome with limited vegetative branching, resulting in small clones of about 30‐cm‐tall stems with multi‐flowered racemes. The peak season of the 2‐ to 2.5‐cm‐long, purplish‐blue flowers is mid‐June. Main pollinators are insects with long tongues, such as bumblebees (Apidae). As for most members of the mint family (Lamiaceae), however, northern dragonhead may also be self‐compatible (Milberg & Bertilsson, 1997). The species' generation time is approximately 15 years (Solstad et al., 2021). An average fruit set rate of 0.27 has been reported for 20 populations in Sweden (Milberg & Bertilsson, 1997). Seeds are dry nutlets lacking modifications for long‐distance dispersal and, hence, most likely are passively dispersed (Kyrkjeeide et al., 2020; Solstad et al., 2021).

Our selection of northern dragonhead as the target species for this study was based on four aspects: (1) The plant's biology as insect‐pollinated and mainly outcrossing (Milberg & Bertilsson, 1997). This is to avoid the complicating aspects of wind‐pollination, polyploidy, and extensive selfing. (2) The abundance availability of specimens through time and space in Norwegian herbaria. (3) The availability of a 96 SNP microfluidic array for northern dragonhead (Kleven et al., 2019), which had already been used for analyzing contemporary samples from Norway (Kyrkjeeide et al., 2020). And, (4) the species' high level of priority in Norway, being threatened by habitat loss. Kyrkjeeide et al. (2020) added a genomic level to the monitoring regime of northern dragonhead in Norway. They found that only two of the revealed genetic groups were covered by the demographic monitoring and conservation efforts at the time.

An exploratory aspect of this study includes testing the performance of a microfluidic array for SNP genotyping, which has been developed based on genomic data from modern specimens of northern dragonhead, on up to 200 years old herbarium specimens of the species. Using this approach, we wanted to study the genetic diversity in northern dragonhead both back in time (i.e., prior to 1950) and across space (mainly in Norway but also beyond). More specifically, we wanted to assess whether the overall and regional genetic diversity of northern dragonhead in Norway has changed. Performing environmental niche modelling (ENM) on occurrence records of northern dragonhead in Norway, we wanted to reveal which abiotic factors may be limiting its distribution and whether there are areas in which northern dragonhead do not occur today that may represent suitable habitats.

## 2 MATERIALS AND METHODS

### 2.1 Materials

**FIGURE 1:**
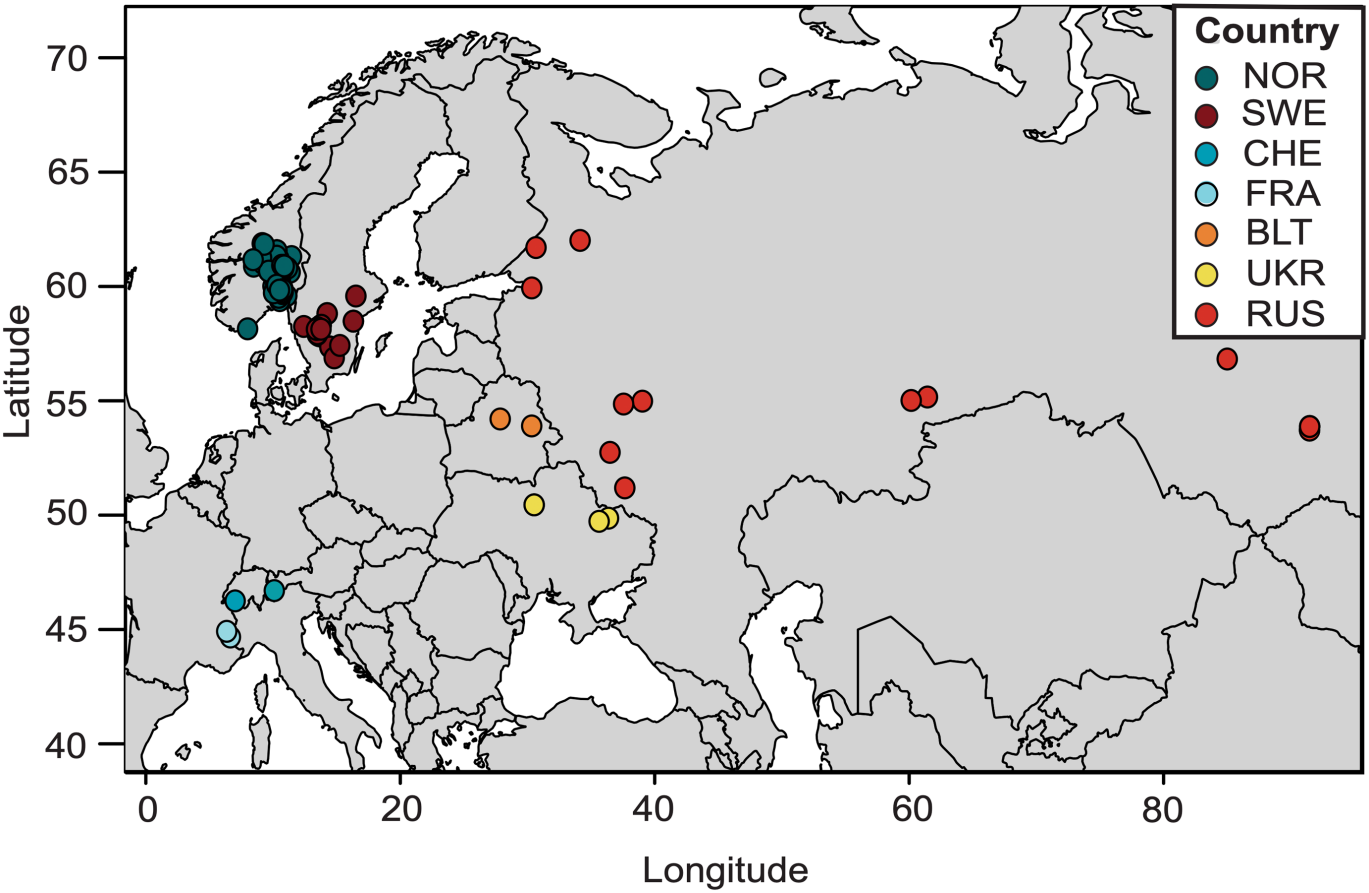
Map of Europe displaying the origins of all 130 included herbarium specimens of *Dracocephalum ruyschiana*. Each point represents a specimen colored according to the country of origin (see inset legend). NOR, Norway; SWE, Sweden; CHE, Switzerland; FRA, France; BLT, Belarus; UKR, Ukraine; RUS, Russia.

For the present study, we sampled 130 herbarium specimens of northern dragonhead collected between 1820 and 2008 (Table S1). The majority of specimens originate from Norway, but we also included 25 specimens from Sweden, 12 from Russia, four from Ukraine, and two from each of the countries Belarus, Switzerland, and France (Figure 1). We additionally included published SNP data from 355 contemporary Norwegian samples of northern dragonhead from 43 different sites (Kyrkjeeide et al., 2022).

### 2.2 DNA extraction and genotyping

All manipulations of the herbarium specimens postsampling were performed within the NTNU University Museum's dedicated, positively pressurized, paleo‐genomics laboratory. About 0.5 cm^2^ of leaf material was removed from each herbarium specimen using clean forceps and placed directly into a microfuge tube. The leaf material was pulverized with two Qiagen 3 mm tungsten carbide beads in a Qiagen TissueLyser LT at 50 Hz for 2 min. We extracted DNA using the DNeasy® Plant Mini Kit (Qiagen) with modifications from the manufacturer's instructions as described by Martin et al. (2014). We incubated the samples for 15 min at 37°C prior to spinning during the elution step. All extractions were performed using UV‐sterilized equipment, and blank samples were always included to monitor for contamination. We measured DNA yield for 116 of the 130 herbarium samples using the Qubit dsDNA BR Assay Kit (Thermo Fisher Scientific, MA, USA), following the manufacturer's protocol. The DNA integrity was evaluated for the same 116 samples through agarose gel electrophoresis. The brightest band on the gel was regarded as an approximation for the sample's mean DNA fragment length. All samples were genotyped using the 96 × 96 SNP array developed by Kleven et al. (2019). The samples were genotyped on a Fluidigm EP1 instrument (Fluidigm Corporation, San Francisco, USA) according to the manufacturer's protocol and scored using the Fluidigm SNP genotyping analysis software v. 4.5.1 (https://www.fluidigm.com/software). Positive and negative controls were included.

We excluded SNPs with more than 10% missing data across all herbarium samples. For each genotyped sample, we subsequently calculated the call rate (CR) and the proportion of successfully genotyped loci. The relationships between specimen collection year, CR, and DNA concentration were estimated using the Pearson's correlation test implemented in R (R Development Core Team, 2021).

### 2.3 Genetic structure and diversity

The contemporary samples from Kyrkjeeide et al. (2022) are herein referred to as *the modern group*, whereas the herbarium samples are referred to as *the historical group*. We established two main datasets, NOR and GLOB, which we analyzed in their entirety or as subsets for assessing genetic structure and diversity. The NOR dataset included SNP data from Norwegian samples only, both historical and modern, and the former only from herbarium specimens collected prior to 1950 (*N* = 76). Due to incomplete overlap between historical and modern sampling sites (some even representing extinct populations), which complicated direct temporal comparisons of populations, we grouped samples into the geographical regions that correspond to the distinct genetic groups discovered by Kyrkjeeide et al. (2020). Regions containing a sole historical sample were included only in our analyses of genetic structure. The GLOB dataset included SNP data from all 130 genotyped herbarium specimens, independent of collection year (83 Norwegian and 47 extra‐Norwegian; Figure 1). The NOR and GLOB SNP datasets used in the present study are publicly available at DRYAD (https://doi.org/10.5061/dryad.c59zw3r8g).

The genetic structure within northern dragonhead was assessed using ParallelStructure v. 2.3.4 (Besnier & Glover, 2013; Pritchard et al., 2000), an R‐based implementation of the common STRUCTURE algorithm, on XSEDE at CIPRES Science Gateway v. 3.1 (Miller et al., 2011). For the NOR dataset, we tested *K* from 1 to 40, with 20 replicates for each value of *K*. For the GLOB dataset, we performed three different runs including (1) all samples, (2) only extra‐Norwegian samples, and (3) only 2–4 individuals from each country for a reduced and balanced sampling. For all three runs with GLOB, we tested *K* from 1 to 10, with 20 replicates for each value of *K*. Calculation of the optimal number of clusters and visualization of the results were obtained using *find.cluster* (R package *adegenet* v. 1.3–1; Jombart, 2008) and StructureSelector (Li & Liu, 2018), which implements the Puechmaille method of optimization (Puechmaille, 2016) in addition to calculating *Ln Pr(X|K)* and Δ*K* (Evanno et al., 2005).

For both the NOR and GLOB datasets, genetic structure and differentiation was evaluated through the use of discriminant analyses of principal components (DAPC; Jombart et al., 2010). Groups were defined a priori according to geography; by region for NOR and by the country for GLOB. For NOR, we also tested a priori grouping by historical versus modern samples. We additionally calculated the fixation index (*F*_ST_) for NOR using the R function *stamppFst* (package StAMPP; Pembleton et al., 2013), after converting our data into a *genlight* object. Pairwise *F*_ST_ values were calculated between the overall historical and modern groups, and between regional areas within the historical and modern groups, respectively, in addition to between the two age groups within the same regional areas. The 95% confidence interval was estimated using 1000 bootstraps.

For NOR, after transforming our data to a *genind* object, we calculated observed (*H*_O_) and expected heterozygosity (*H*_E_), and the inbreeding coefficient (*F*_IS_) using the *basic.stats* function in R. We used the R *summary* function (*adegenet* package) to obtain the number of alleles, *isPoly* for the number of polymorphic loci, and *private_alleles* (R package *poppr*; Kamvar et al., 2014) for the number of private alleles. All measures of genetic diversity were calculated for the historical and modern regions, and averaged across all regions within each age group. In order to evaluate the effect of the sample size on the reported values, we subsampled modern regions down to equal sample size as their equivalent historical region. The subsampling was done randomly in 10 replicates using the R function *sample*, and diversity measures were recalculated in each run. Subsequently, we averaged across all replicates and calculated the standard deviation (SD). To visually explore the potential change in genetic diversity over time, we plotted the estimated *H*_E_ of historical and modern regions against the oldest and youngest collection year, respectively. The subsample averaged *H*_E_ (and SD) was used for regions with uneven historical and modern sample size. We additionally plotted the individual proportion of heterozygous loci (PHt) against the sample collection year within each region.

For GLOB, genetic diversity was estimated as the proportion of heterozygous loci per individual (PHt). We calculated the number of polymorphic loci for the individual countries utilizing the *adegenet* function *isPoly*. To evaluate whether the GLOB genetic diversity was affected by distance from the SNP array's source population (Norway), we estimated the Pearson's correlation between PHt and the sample localities' distance from Oslo (59.9138 N, 10.7387E), an approximate centre of the SNP array's source populations.

### 2.4 Environmental niche modeling

Species occurrence records for preserved specimens of Norwegian northern dragonhead were downloaded from the GBIF (10.15468/dl.748g3v, accessed via GBIF.org on 2021‐03‐13). We added coordinates for GBIF‐IDs lacking this information according to locality information and its precision (Table S2). Prior to analyses, we removed occurrence records that were clearly originating from a garden or otherwise represented a cultivar (Table S3).

Modeling the species' distribution was based on a final dataset of 4092 species occurrence records. The environmental niche modelling of northern dragonhead was based on three variables: mean temperature of the warmest quarter (i.e., mean summer temperature; MST), mean annual precipitation (MAP), and precipitation seasonality (coefficient of variance of monthly precipitation; PS). These are the same variables that, according to Speed and Austrheim (2017), represent the majority of uncorrelated variation in the total bioclimatic space of Norway. The climatic data were downloaded from WorldClim (Fick & Hijmans, 2017) at 1‐km resolution (Figures S1–S12). We created background data by sampling 1000 random occurrence points across Norway, weighted by the distribution of occurrence data of all vascular plants in Norway. The *sdm* R package (Naimi & Araújo, 2016) was used to run several different distribution models: generalized linear model (GLM), generalized additive model (GAM), random forest (RF), gradient boosting machines (GBM), mixture discriminant analysis (MDA), flexible discriminant analysis (FDA), and boosted regression trees (BRT). We cross‐validated each model with five replicate runs. The results and predictions were subsequently averaged across all methods and replicates using a weighted average based upon the model area under the curve (AUC). The variable importance and response curves of northern dragonhead were estimated prior to modeling its environmental niche across Norway.

## 3 RESULTS

### 3.1 SNP genotyping performance

**FIGURE 2:**
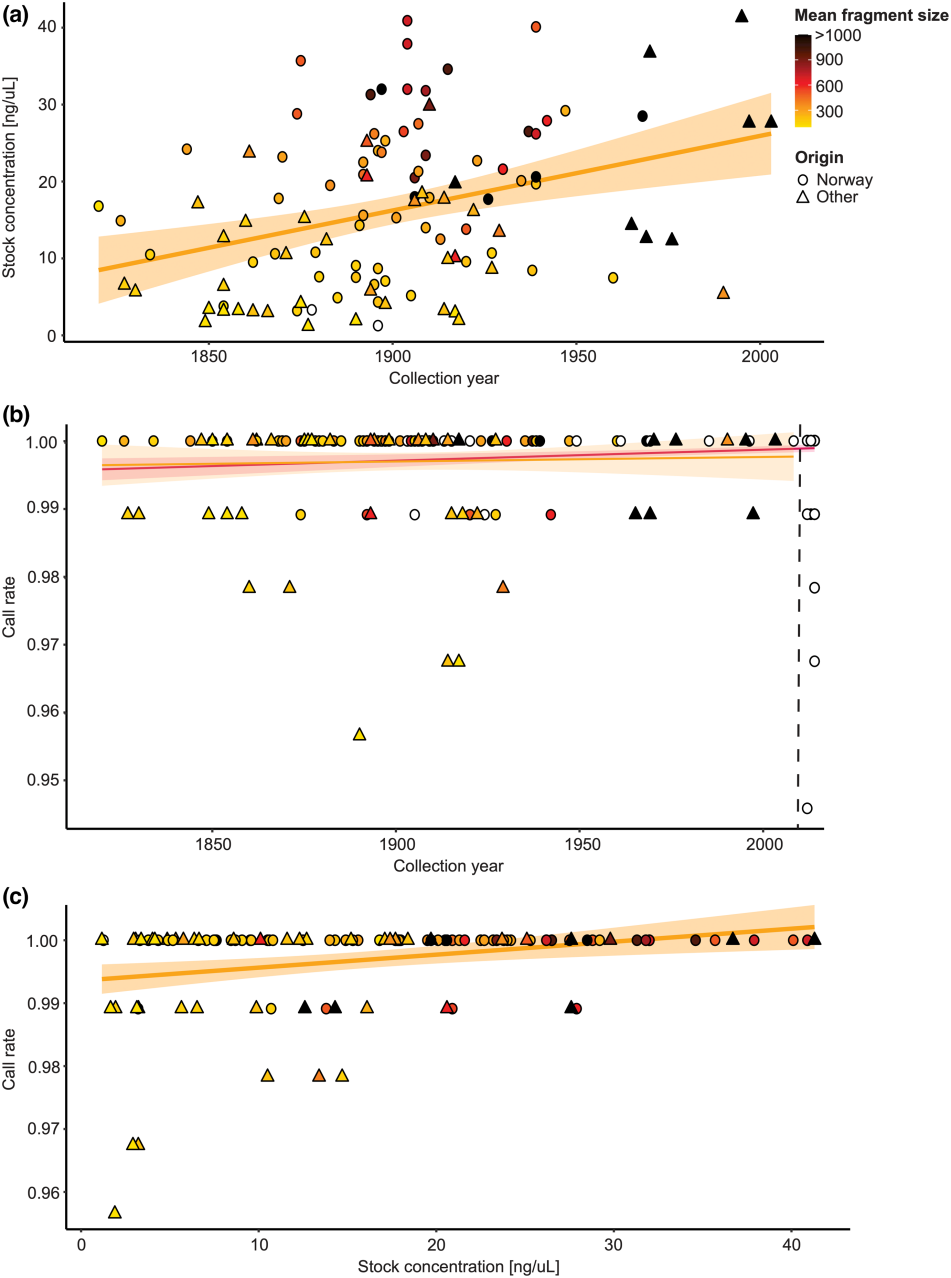
Correlation plots for DNA quality (as stock concentration and mean fragment size), call rate (CR; the proportion of successfully genotyped SNPs per sample), and age of the *Dracocephalum ruyschiana* samples (given as collection year). The orange lines represent the average overall historic samples and the orange zones the 95% confidence interval. Each symbol represents an individual sample, the shape of its geographical origin, and the color of its mean DNA fragment size (bp) based on gel electrophoresis (white = no data). (a) DNA quality: Correlation between specimen collection year and Qubit DNA concentration for 113 historical *D. ruyschiana* specimens. (b) SNP genotyping performance: correlation between specimen collection year and CR for 127 historical and 355 modern *D. ruyschiana* specimens, displayed to the left and right of the stippled line, respectively. The pink line represents the average over both historical and modern samples and the pink zone is 95% confidence interval. (c) Correlation between Qubit DNA stock concentration and CR for 116 historical *D. ruyschiana* specimens.

For the herbarium specimens, the DNA stock concentration varied from 1.17 to 41.30 ng/μl, with a mean of 16.11 ng/μl. The DNA stock concentration and specimen's collection year were positively correlated with a Pearson's correlation coefficient of *R* = 0.35 (*p* < .001; Figure 2a). Due to a technical error, four SNPs had missing data for more than 10% of all genotyped samples. We removed these prior to analyses. Across all historical samples and the remaining 92 SNPs screened, the mean call rate (CR) was 99.71%, ranging from 95.65% to 100%. In comparison, the modern samples had a mean CR of 99.89%, varying from 94.57% to 100%. When separating historical Norwegian versus extra‐Norwegian samples, the mean CR was 99.91% and 99.35%, respectively. Considering only historical specimens, there was no significant correlation between CR and collection year (Pearson's correlation coefficient; *R* = 0.04 *p* = .68; Figure 2b, orange line), while there was a slight positive correlation when combining the historical and modern samples (*R* = 0.15 *p* < .001; Figure 2b, pink line). Comparing CR and DNA stock concentration among all historical samples resulted in a weak positive correlation, *R* = 0.28 (*p* = .002; Figure 2c).

### 3.2 Genetic structure and diversity — Norwegianscale

**FIGURE 3:**
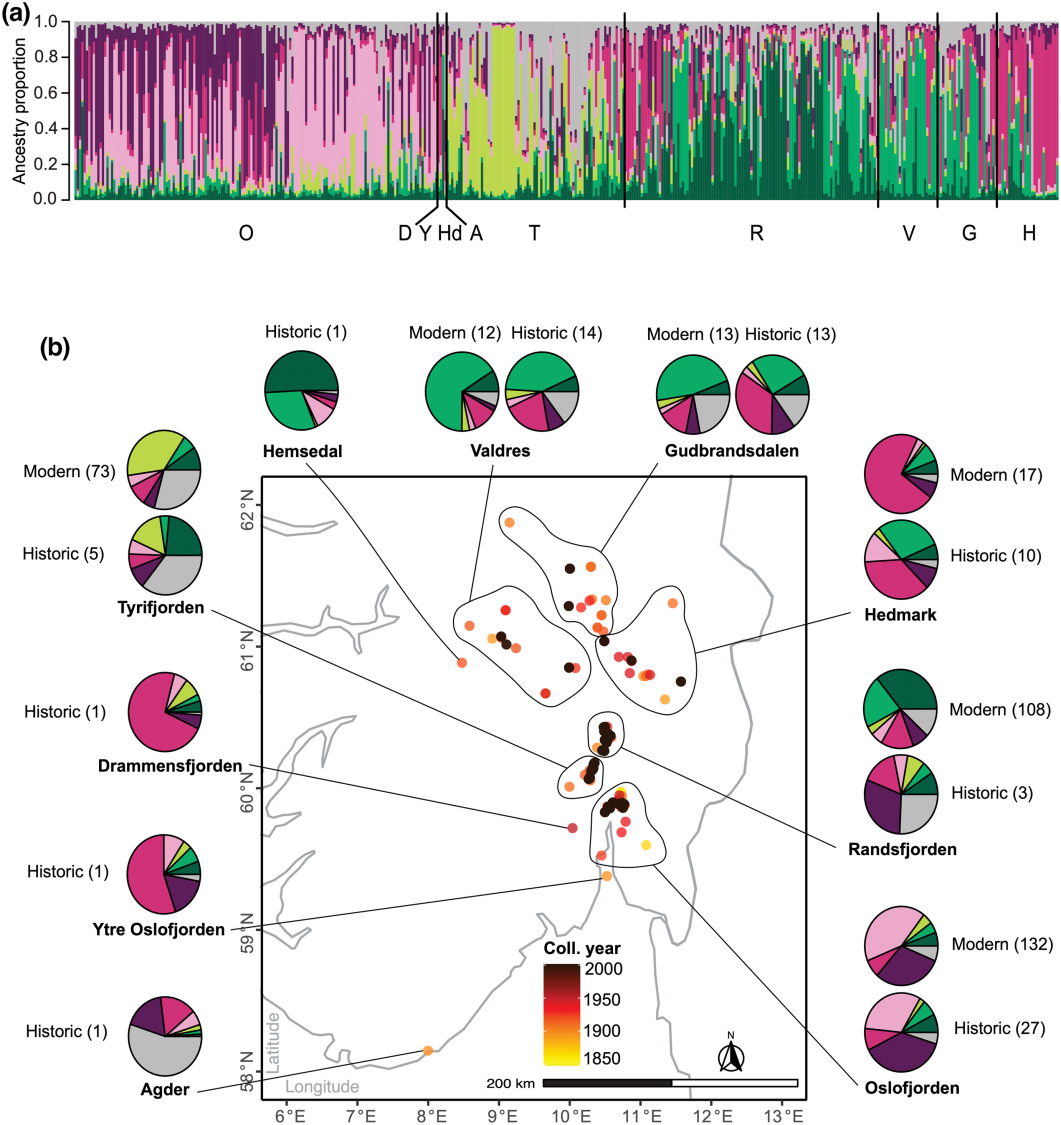
Structure results at *K* = 7 for our Norwegian *Dracocephalum ruyschiana* SNP data (NOR dataset). (a) Vertical bars represent individuals and their ancestry proportion of each genetic cluster, the latter displayed by the size of the color segment. Samples are sorted by municipalities within the larger geographic areas (regions) and subsequently by modern and historical samples, respectively. A, Agder; B, Buskerud; D, Drammensfjorden; G, Gudbrandsdalen; H, Hedmark; O, Oslofjorden; Y, Ytre Oslofjorden; R, Randsfjorden; T, Tyrifjorden; V, Valdres. (b) Pies represent the average ancestry proportions (same coloring scheme as in a) of all historical and modern Norwegian regions. The number of individuals in each temporal region is in parentheses. Points on the map of southeastern Norway represent sample localities. The color of each point indicates the sample's collection year; modern samples are colored black and historical yellow to red.

For the STRUCTURE analysis conducted on the NOR dataset (*N* = 431), the optimal number of genetic clusters varied depending on the applied optimization method. The mean log posterior *ln P(K)* was found to continuously increase with increasing *K* and reached the highest value for *K* = 24 (Figure S2). We found the highest value of Δ*K* for *K* = 2, although *ln P(K)* was low at *K* = 1. Highest MedMed *K*, MedMean *K*, and MaxMean *K* were observed for *K* = 7, whilst MaxMed *K* was highest for *K* = 8. Using *find.cluster*, the lowest BIC value was found between *K* = 5 and *K* = 8 (Figure S3). Under the most frequently inferred number of clusters (*K* = 7), when sorting samples according to predefined regions, six of the clusters largely corresponded to the regional areas: Hedmark, Oslofjorden (east and west), Randsfjorden, Tyrifjorden, and Valdres‐Gudbrandsdalen (Figure 3a). The other regional areas that appeared admixed for all *K*s (i.e., Agder, Drammensfjorden, Hemsedal, and Ytre Oslofjorden; Figure S4) were excluded from downstream analyses because each contained only a single sample. When dividing our STRUCTURE results into historical and modern groups, most of the regional areas displayed similar genetic structures through time (Figure 3b). The greatest temporal change in ancestry proportions was observed for Randsfjorden, whereas the least change through time was observed for Oslofjorden.

**FIGURE 4:**
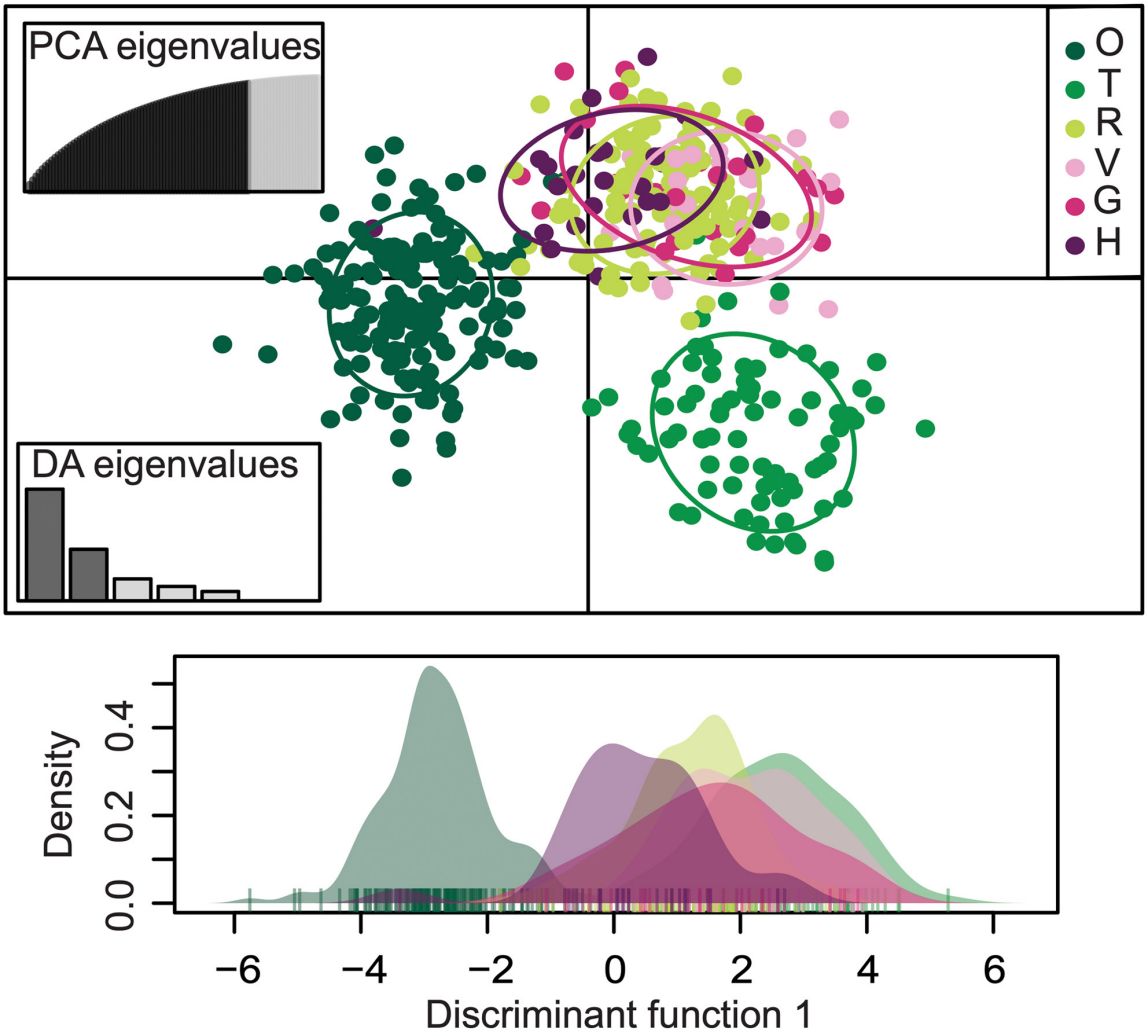
DAPC results for our Norwegian *Dracocephalum ruyschiana* SNP data (NOR dataset), including 83 historic and 355 contemporary specimens (not indicated). Groups were a priori defined according to the geographic regions of Kyrkjeeide et al. (2020; see inset legend). In the scatter plot (upper), points represent individuals, and the different colors and inertia ellipses show the predefined groups. The bar plot with DA eigenvalues displays the proportion of genetic information explained by each consecutive discriminant function. The density plot (lower) presents the distribution of each predefined group on the first discriminant function, in their respective colors.

The DAPC results corroborated the separation of Oslofjorden and Tyrifjorden, respectively, from the remaining regions, when a priori grouping our samples according to the regional areas (Figure 4). The first and second DA explained 53.4% and 24.7% of the genetic variation, respectively. A priori grouping of the specimens by age (historical vs. modern) for the DAPC analysis resulted in overlapping density curves along the first axis (Figure S5). Also, in terms of *F*‐statistics, we observed larger genetic divergence across space than through time. The overall *F*_ST_ value between the historical and modern groups indicated a very low overall level of temporal genetic divergence (i.e., 0.003 with 95% CI from 0.002 to 0.004). Pairwise comparisons of regions (in both time and space) yielded generally low *F*_ST_ values, but all 95% confidence intervals were above zero (Table S4). The largest temporal genetic divergence was observed for Randsfjorden (*F*_ST_ = 0.033) and Tyrifjorden (*F*_ST_ = 0.028), which both also displayed a decline in spatial divergence over time. This decline in *F*_ST_ was only significant for Randsfjorden (Table 1). Values estimated for Randsfjorden and Tyrifjorden should, however, be interpreted with caution due to low historical sample sizes (*N* = 3 and *N* = 5, respectively). The genetic differentiation between Gudbrandsdalen and Hedmark, on the other hand, increased significantly over time, and represented the highest value among all modern pairwise comparisons (*F*_ST_ = 0.042).

**TABLE 1:** Genetic differentiation within Norwegian *Dracocephalum ruyschiana* (NOR), in both time and space.

	Oslofjorden	Tyrifjorden	Randsfjorden	Valdres	Gudbrandsdalen	Hedmark
Oslofjorden	0.006	0.022	0.012*	0.029	0.027	0.027
Tyrifjorden	0.036	0.028	0.016*	0.030	0.027	0.040
Randsfjorden	0.034**	0.061**	0.033	0.019	0.019*	0.023*
Valdres	0.028	0.037	0.042	0.012	0.032	0.036
Gudbrandsdalen	0.027	0.044	0.044**	0.017	0.025	0.042**
Hedmark	0.023	0.050	0.049**	0.021	0.019*	0.022

*Note*: The fixation index values (*F*_ST_) represent pairwise comparisons of either different regional areas in historical times (yellow, lower triangle) or modern times (orange, upper triangle), or between the modern and historical groups within the same regional area (white, diagonal). The *F*_ST_ values that have changed significantly are marked with an asterisk. Only one asterisk means that the value is lower than that of the other age group, and two asterisks means it is higher than that of the other age group. The 95% confidence intervals are displayed in Table S4.

**FIGURE 5:**
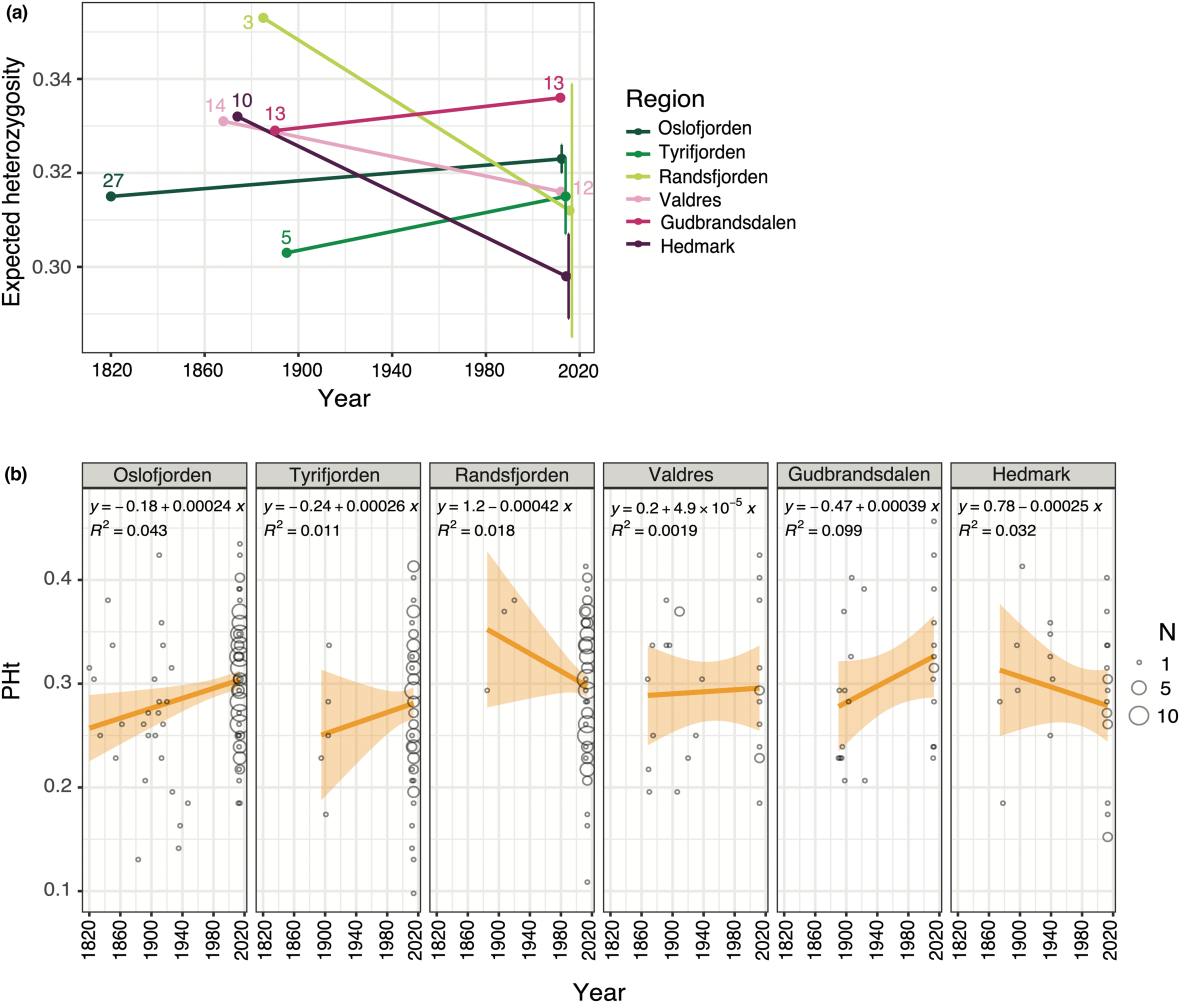
Heterozygosity levels in *Dracocephalum ruyschiana* across time (NOR dataset). (a) Expected heterozygosity (*H*_E_) across time within separate regions, represented by different colored points and lines (see inset legend). The colored numbers above points correspond to the number of samples on which H_E_ estimates were based on. Points with error bars represent modern regions that were subsampled to equal sample size as their equivalent historical region (the point represents the mean across 10 replicated runs and the error bars the standard variation). Along the time axis, historical estimates are according to the oldest sample and modern estimates according to the youngest sample. (b) Individual's proportion of heterozygosity (PHt) in *D. ruyschiana* presented as a function of collection year within separate regions. The size of each point represents the number of individuals (N). The orange line is the regression line, and the orange zone is the 95% confidence interval.

The genetic diversity (*H*_E_) averaged over all regions was slightly higher in the historical (0.327, SD ± 0.017) compared to that in the modern group (0.316, SD ± 0.013), both with positive *F*_IS_ values (Table 2). The average inbreeding coefficient decreased by 0.045 over time, from *F*_IS_ = 0.107 (SD ± 0.056) in the historical to *F*_IS_ = 0.062 (SD ± 0.019) in the modern group. Looking at a finer scale, *H*_E_ ranged from 0.303 to 0.353 for the historical regions and 0.299–0.336 for the modern (Table 2). The direction of change in heterozygosity (*H*_E_ and PHt) across time varied between regions (Table 2; Figure 5). The largest difference in *H*_E_ over time was found in Hedmark and Randsfjorden, which had decreased by 0.033 and 0.039, respectively. For Gudbrandsdalen, Oslofjorden, and Tyrifjorden, we observed the lowest change in *H*_E_, with an increase in only 0.004 to 0.007. The same three regions also displayed the highest increase (0.071 to 0.101) in *F*_IS_ values (Table 2). The number of alleles and polymorphic loci was largest within the modern group (Table 2). No private alleles were found for the historical versus modern group, and no specific region contained private alleles compared to the other regions within the same age group. We did, however, observe private alleles when comparing the historical and modern samples within single regions (Table 2). The highest amounts of private alleles were found in modern Randsfjorden (25) and Tyrifjorden (21) compared with their respective historical regions, likely a result of uneven sample sizes between the age groups. The subsampling of modern samples to historical sample sizes displayed that Randsfjorden had the largest standard deviation for all diversity measures (Table S5; Figure 5a). The inbreeding coefficient (*F*_IS_), averaged across all replicates, was 0.085 (SD ± 0.032) for Hedmark, 0.052 (SD ± 0.012) for Oslofjorden, 0.073 (SD ± 0.092) for Randsfjorden, and 0.110 (SD ± 0.067) for Tyrifjorden.

**TABLE 2:** Genetic diversity within Norwegian *Dracocephalum ruyschiana* (NOR), across time and space.

Group: region	*Nind*	*Nallele*	*Npoly*	*HO*	*HE*	*FIS*	Miss (%)
Historical							
Oslofjorden	27	181 [0]	89	0.267	0.315	0.153	0.04
Tyrifjorden	5	161 [0]	69	0.256	0.303	0.156	0.22
Randsfjorden	3	159 [0]	67	0.348	0.353	0.015	0
Valdres	14	179 [6]	87	0.292	0.331	0.117	0.16
Gudbrandsdalen	13	181 [3]	89	0.284	0.329	0.137	0.08
Hedmark	10	176 [6]	84	0.311	0.332	0.065	0.22
Mean	12	172.83	80.83	0.293	0.327	0.107	0.12
SD	8.53	10.13	10.13	0.033	0.017	0.056	0.09
Modern							
Oslofjorden	132	184 [3]	88	0.306	0.322	0.052	0.12
Tyrifjorden	73	182 [21]	89	0.281	0.307	0.085	0.13
Randsfjorden	108	184 [25]	91	0.298	0.314	0.052	0.12
Valdres	12	174 [1]	82	0.294	0.316	0.07	0
Gudbrandsdalen	13	180 [2]	88	0.324	0.336	0.035	0
Hedmark	17	176 [6]	84	0.275	0.299	0.081	0
Mean	59.17	180	87	0.296	0.316	0.062	0.06
SD	52.94	4.2	3.35	0.018	0.013	0.019	0.07

*Note*: Number of individuals (*N*_**ind**_), alleles (*N*_**allele**_), polymorphic loci (*N*_**poly**_), observed (*H*_**O**_) and expected heterozygosity (*H*_**E**_), inbreeding coefficient (*F*_**IS**_), and percentage of missing data (%) within separate regions of the historical and modern group. The number of private alleles for similar regions (historical vs. modern) is provided within square brackets. Similar measures for subsampled modern regions are provided in Table S4.

### 3.3 Genetic structure and diversity — Global scale

Results from the STRUCTURE analysis on the full GLOB dataset varied with regard to the number of optimal genetic clusters depending on the applied optimization method (Figure S6). The mean log posterior, *ln P(K)*, increased until *K* = 3 and made a drop before increasing to its maximum at *K* = 7. We found the highest value of Δ*K* under *K* = 2, although *ln P(K)* was low at *K* = 1. We found the highest MedMed *K*, MedMean *K*, MaxMean *K*, and MaxMed *K* for *K* = 4. Using *find.cluster*, we observed the lowest BIC value between *K* = 2 and *K* = 6 (Figure S7). At *K* = 2, Norwegian samples separated from the remaining European samples (Figure S8). At *K* = 4, Swedish samples formed their own group while French and Swiss samples displayed mixed ancestry from the Norwegian and Swedish clusters (Figures S8–S8). Further increasing *K* led to a higher degree of admixture, mainly within Norway, but also to some degree within Sweden, Switzerland, and France (Figure S8). Belarus, Russia, and Ukraine, on the other hand, consistently formed a single cluster. For the other two STRUCTURE analyses, excluding Norwegian samples (Figures S10–S11) and balancing sampling across countries (Figures S12–S13), we observed that *ln P(K)* increased until *K* = 4 and *K* = 3, respectively. For increasing values of *K*, the *ln P(K)* continued to decrease. For both these analyses, the highest value of Δ*K* was found under *K* = 2, whereas MedMed *K*, MedMean *K*, MaxMean *K*, and MaxMed were highest at *K* = 3.

**FIGURE 6:**
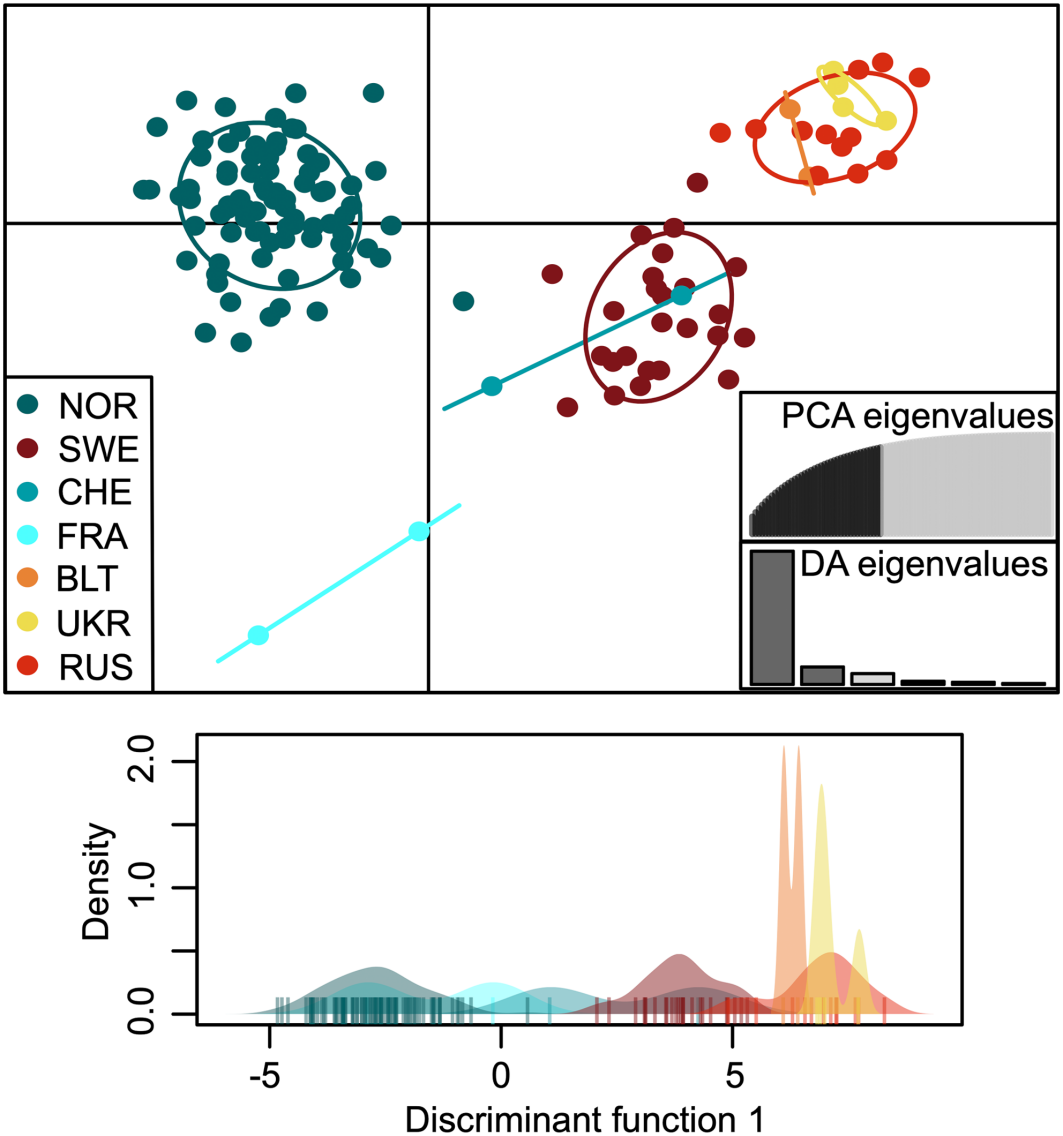
DAPC analysis for 130 European *Dracocephalum ruyschiana* specimens originating from seven different countries (GLOB dataset). In the scatter plot (upper), points represent individuals, and the different coloring and inertia ellipses show the predefined groups (see inset legend). The bar plot with DA eigenvalues displays the proportion of genetic information explained by each consecutive discriminant function. The density plot (lower) presents the distribution of each predefined group on the first discriminant function, in their respective colors.

When a priori grouping the herbarium specimens by geography (i.e., by country), the first and second DA explained 59.3% and 40.7% of their total genetic variation, respectively (Figure 6). The DAPC analysis separated the Norwegian population from the remaining Eurasian countries. We also found the highest genetic diversity within the Norwegian samples, measured as individual proportions of heterozygosity (PHt) and the number of polymorphic loci (Figure S14a, b). The individual PHt decreased significantly with increasing distance from Norway (*R* = −0.49, *p* = 5.69 × 10^−9^; Figure S14c).

### 3.4 Environmental niche modeling

**FIGURE 7:**
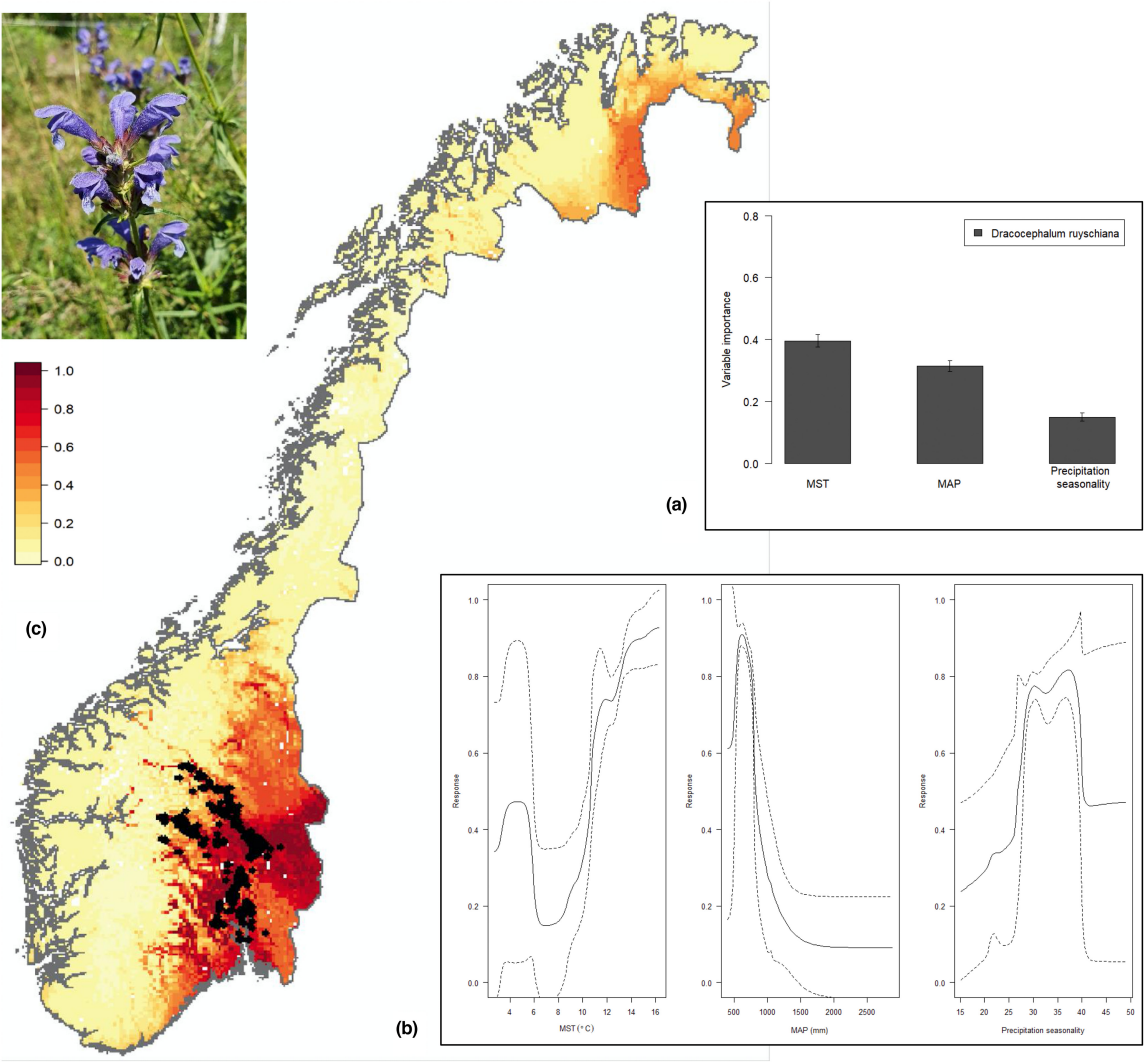
(a) Variable importance in spatial predictions for *Dracocephalum ruyschiana* across Norway based on 4092 species occurrence records downloaded from the Global Biodiversity Information Facility (GBIF). The three variables represent the three main axes of bioclimatic variation within Norway: Mean temperature of the warmest quarter (MST), Mean annual precipitation (MAP), and Precipitation seasonality (PS; Speed & Austrheim, 2017). (b) Response curves of climatic suitability for *D. ruyschiana* against the three selected bioclimatic variables. Solid and dashed lines show mean and standard errors, respectively. (c) Spatial prediction of *D. ruyschiana* across Norway based on ecological niche modeling of 4092 occurrence records. Darker red color represents higher, and lighter yellow color represents lower niche suitability. The black points display occurrence records.

Across all the replicated environmental niche models, the mean AUC was 0.95 (SD ± 0.03). The relative variable importance was highest for mean summer temperature (MST; 0.40, SD ± 0.02), followed by mean annual precipitation (MAP; 0.31, SD ± 0.02), and lowest for precipitation seasonality (PS; 0.15, SD ± 0.01; Figure 7a). Based on the response curves, climate suitability for northern dragonhead increased with higher temperatures, MST > 10°C, and decreased with increased precipitation, MAP > 500 mm (Figure 7b). After averaging over all models, the model predicted the greatest niche suitability in southeastern Norway (Figure 7c). Potentially suitable, but unoccupied, niche space was predicted around Trysil and in the lowland valleys east of the Trondheimsfjord, among other areas in western and northern Norway (Figure 7c).

## 4 DISCUSSION

Maintenance of genetic diversity is a central aim of species conservation, given its positive role in a species' performance and survival in a changing environment (Lande & Shannon, 1996). In this study, we have assessed changes in genetic structure and diversity across space and through time in northern dragonhead, a charismatic flowering plant that has experienced a drastic population decline and habitat loss in Europe. We have added a temporal level to the monitoring of northern dragonhead in Norway using an SNP array technology on herbarium specimens. To identify which abiotic factors may limit its distribution and whether there are additional areas with suitable habitats, we have used sample metadata and observational occurrence records to model the species' environmental niche.

### 4.1 Microfluidic SNP genotyping performance on herbarium specimens

#### 4.1.1 SNP genotyping performance through time

All the included herbarium specimens of northern dragonhead yielded DNA of a quality suitable for SNP genotyping. Even though the DNA stock concentration decreased with specimen age (Figure 2a), the negative correlation was weaker than expected. Previous time‐series studies of herbarium samples have shown that both molecular weight (DNA fragment length) and stock concentration decreased with time since collection (see Raxworthy & Smith, 2021, and references therein). The rate of decrease in molecular weight and DNA concentration apparently depends on the samples' history, such as the way it was collected and preserved, and the subsequent storage conditions. In addition, DNA concentration appears to vary among different parts of the specimen, tissue types, and preservation techniques. Indeed, for herbarium specimens, most of the DNA damage appears to occur soon after sampling (i.e., during specimen preparation; Staats et al., 2011). The best practice for preserving plant DNA is assumed to be rapid desiccation under moderate temperatures.

Despite the lower DNA concentration of the extracts from the historical specimens, all samples were successfully SNP genotyped. As for the DNA stock concentration, the call rate (CR) seemed to be surprisingly little affected by time since collection when comparing modern with historical samples (Figure 2b); our historical samples obtained a consistently high CR (mean = 99.71%, min = 95.65%, and max = 100%), which was approximately equal to that of the modern samples (mean = 99.89%, min = 94.57%, and max = 100%). In addition, historical materials of both animals and plants have been successfully genotyped using microfluidic SNP arrays (e.g., Finch et al., 2020; Johnston et al., 2013; Östergren et al., 2021). Compared with our results, however, these authors found much higher differences in the CR between historical and modern samples. Finch et al. (2020), for example, who applied a microfluidic array of 140 SNPs on historical and modern samples of the neotropical tree *Cedrela odorata* and relatives, reported much lower and more variable CR values (0–96%) for the herbarium specimens.

Assuming a positive correlation between DNA stock concentration and CR, which our results on northern dragonhead indicate (Figure 2c), is transferable to other taxa, the results by Finch et al. (2020) suggest that the quality of their DNA extracts (stock concentration and molecular weight) from the historical material are lower. Such a discrepancy in DNA quality between different plant species may be explained by a combination of differences in their inherent biology and preservation techniques and conditions. Tropical trees, like *C. odorata*, typically contain high levels of anti‐predation polyphenolic compounds in their leaves (see Colpaert et al., 2005, and references therein), which may negatively affect the quality and quantity of the extracted DNA (see Aboul‐Maaty & Oraby, 2019, and references therein). However, such secondary compounds have not been hindering previous molecular work on the Lamiaceae, the flowering plant family to which northern dragonhead belongs (see e.g., Bendiksby et al., 2011). Moreover, the fact that our study object occurs in the temperate zone, rather than in the tropics, implies that the specimens studied have been living in a less harsh climate (i.e., moderate temperatures) with better facilities for rapid desiccation. Hence, although the microfluidic SNP array approach was highly successful for northern dragonhead, this may not be the case for historical specimens of species that, for biological reasons, experience faster DNA degradation, or that cannot be desiccated rapidly under moderate temperatures.

#### 4.1.2 SNP genotyping performance across space

For the global dataset (GLOB), our results demonstrate the effect of SNP ascertainment bias (i.e., the selection of loci from an unrepresentative sample of individuals), which shows a systematic deviation from theoretical expectations (Geibel et al., 2021). Since the SNP array was designed based on highly polymorphic SNPs from Norwegian northern dragonhead samples, the allele frequencies were expected to be lower in populations outside Norway. This is apparent from our global measures of genetic diversity, which decrease significantly with increasing geographical distance from Norway (Figure S14c). On the other hand, ascertainment bias is apparently less likely to affect the assignment of individuals to separate populations (Lachance & Tishkoff, 2013). As such, our results indicate that the Norwegian samples are genetically distinct from the examined materials originating from elsewhere in Eurasia (Figures 6, S9). To determine the degree to which they are distinct cannot, however, be estimated based on our current SNP data.

#### 4.1.3 Microfluidic SNP array optimization

The critical step for obtaining informative SNP data lies in the selection of SNP markers and the development of the SNP array itself. Since genetic diversity is often unevenly distributed across space and through time, the SNP data will be biased towards variants present in the samples from which the selected SNPs originate (Geibel et al., 2021). For example, genetic diversity only present in the past will not be recovered by an array designed based on modern material alone. To reduce ascertainment bias and to obtain more precise genetic estimates for a spatiotemporal study, the array of SNPs should be specifically designed to target genetic variation both across space and through time. Moreover, to avoid overestimating levels of genetic diversity, the SNPs should be sampled randomly and not targeted towards the highest level of variation. It should be noted that the SNP array used herein was originally designed for recognition at the level of individuals and not populations (see Kleven et al., 2019), which may have resulted in less distinct population genetic structures and an overall higher level of genetic diversity.

#### 4.1.4 SNP array + herbarium = cost‐ and risk savings

Apart from successfully genotyping historical herbarium specimens, SNP genotyping with microfluidic arrays also offers a cost‐ and time‐efficient method for generating genomic datasets for many samples (von Thaden et al., 2017). This is particularly the case when genomic data for SNP selection is already available (e.g., genome skims or RAD/GBS data). Prior to loading the DNA onto the array, no library preparation is required, and large numbers of samples can be processed simultaneously. Furthermore, there is no need for extensive bioinformatic skills, the raw data require less storage space, and the computational time for filtering and processing the data is comparably short. Hence, for processing many samples for genetic monitoring purposes, including historical ones with variously degraded DNA for temporal monitoring, microfluidic SNP genotyping appears to be a promising method of choice due to reduced overall cost and labour as compared to other currently available methods.

Working with historical specimens provides several key benefits compared to using contemporary material alone. As demonstrated by our study, incorporating historical specimens, which could even include extinct populations, enables the assessment of genetic change over time. Such knowledge is clearly relevant for making sound conservation priorities. Often expert‐validated, herbarium collections create a solid basis and enlarge contemporary datasets of other researchers to conduct genetic studies on historical material and temporal processes, including genetic variation within taxa with challenging identification. Borrowing specimens from other herbaria reduces the health risks and costs associated with traveling and fieldwork. This is especially true within ravaged areas or remote localities. Lastly, sampling from herbarium collections eliminates the ethical dilemma of exposing red‐listed or protected species to further threats, in addition to limiting sample logistics and potential bureaucracy overall.

### 4.2 Northern dragonhead through time and across space

#### 4.2.1 Temporal genetic stasis at species level?

By comparing our obtained SNP data from herbarium specimens with modern SNP data of Kyrkjeeide et al. (2020), we found no indications of substantial temporal changes in the overall genetic structure or diversity of northern dragonhead within Norway, despite the reduction in population size in recent times. Both age groups displayed the similar geographical distribution of genetic variants (Figures 3, 4), with close to no temporal genetic divergence (*F*_ST_ = 0.003) or changes in levels of heterozygosity through time (historical mean *H*_E_ = 0.327 and modern mean *H*_E_ = 0.316). We did record a small decrease in the overall inbreeding coefficient over time, from *F*_IS_ = 0.107 in the historical to *F*_IS_ = 0.062 in the modern group. A decrease in *F*_IS_ (but still *F*_IS_ >0) could be an indication of overall less effects of genetic drift or a higher degree of outcrossing compared with historical times. It should, however, be mentioned that the standard deviation for the obtained overall *F*_IS_ was relatively large for the historical group (SD ± 0.056). Moreover, our current data may not be suitable for robustly inferring a reduction in population size, due to ascertainment bias of the SNP array. The SNP array we have used is based on modern material alone and would not have recovered genetic diversity only present in historical samples.

Previous studies utilizing SNP data to investigate changes in genetic diversity through time have found more pronounced, temporal differences within their target species (e.g., Gauthier et al., 2020; Östergren et al., 2021). Gauthier et al. (2020) demonstrated genetic erosion within two species of Finnish butterflies over a time span of 100 years, using 2742 SNPs; not strictly comparable to our study, as improved precision is expected with increasing numbers of SNPs (Bradbury et al., 2015). Östergren et al. (2021), on the other hand, detected temporal homogenization within Atlantic salmon (*Salmo salar* L.) over approximately 100 years with only 82 SNPs (vs. 92 in the present study). Species with shorter generation turnover tend to have higher rates of temporal genetic change, presumably because more frequent genome replication leads to more replication errors per unit time (Thomas et al., 2010). The comparatively low change in temporal genetic structure or diversity of northern dragonhead in Norway may be impacted by the species having a longer generation turnover (ca. 15 years; Solstad et al., 2021) than for instance salmon (ca. 6 years; Östergren et al., 2021). Moreover, interpreting *F*_ST_ may be challenging as the measure varies depending on the real genetic variation and the selected markers (Hedrick, 2005). The SNP markers applied in our study were specifically developed on a particular set of Norwegian populations of *D. ruyschiana*. Still, comparable results on genetic differentiation have been reported from *D. austriacum*, a close relative of *D. ruyschiana* in the Czech Republic and Slovakia (Dostálek et al., 2010).

A seemingly unchanged distributional range in Norway and limited dispersal may also have contributed to the observed temporal genetic “stasis” within northern dragonhead. Despite a reduction in suitable habitats over the last 150 years, observational data indicate that the overall distributional range of northern dragonhead in Norway has remained largely intact, and that the decline has been mainly local rather than regional (Norwegian Directorate for Nature Management, 2010: Figure 6). Given its pollination syndrome (insect pollination; Milberg & Bertilsson, 1997) and relatively large seeds, the northern dragonhead is primarily an outcrossing species with presumably poor abilities for long‐distance dispersal. Additionally, the landscape topology of Norway, corresponding well with the predefined regional areas used herein (adopted from Kyrkjeeide et al., 2020), likely limits dispersal between regions naturally.

It should be mentioned that isolation by distance (IBD) was shown to be present in modern samples of northern dragonhead in Norway (Kyrkjeeide et al., 2020: Figure 2). Their Mantel test revealed a positive correlation between genetic distance and geographical distance (*R* = 0.56, *p* = .001). The analysis software STRUCTURE, which we have used herein, assumes that markers are not linked and that populations are panmictic (Pritchard et al., 2000). Hence, our STRUCTURE results should be interpreted with caution, as IBD violates the assumption of freely distributed genotypes. In our study, however, also the DAPC results support that genetic variation within historical and modern northern dragonhead is better explained by divergence across space than divergence through time. The DAPC analysis software is a model‐free method based on K‐means clustering of genetic distance and IBD does not violate its assumptions (Jombart et al., 2010).

#### 4.2.2 Minor temporal genetic change at regional level

At the regional scale, the temporal genetic changes were also small. The direction of change, however, varied between regions. For four of the regions (Gudbrandsdalen, Oslofjorden, Tyrifjorden, and Valdres), the inbreeding coefficient decreased over time (Table 2). There was, however, still an excess of homozygosity relative to Hardy–Weinberg Equilibrium (*F*_IS_ > 0), indicative of genetic drift or inbreeding. Surprisingly, the genetic diversity (*H*_E_) within Gudbrandsdalen, Oslofjorden, and Tyrifjorden had increased over time (Figure 5). Given the long generation time of northern dragonhead (15 years), one possible explanation could be a relatively recent increase in gene flow between certain regions compared with previous times. Increased outcrossing could also account for the decrease in genetic divergence between the adjacent regions Oslofjorden and Tyrifjorden over time (historical *F*_ST_ = 0.036 and modern *F*_ST_ = 0.022; see map in Figure 3b). Two regions, Hedmark and Randsfjorden, displayed an increase in *F*_IS_ through time (Table 2). The genetic diversity (*H*_E_) also decreased in both regions, consistent with the loss of rare alleles following genetic drift. As genetic drift proceeds, the genetic divergence is expected to increase, which potentially can explain why the only significant temporal increase in *F*_ST_ values was recorded between Hedmark and its adjacent region, Gudbrandsdalen (Table 1). Randsfjorden, on the other hand, became less differentiated over time despite decreasing genetic diversity and indications of increased drift. Interpretations regarding the temporal change in Randsfjorden should, however, be conducted with caution, due to its low historical sample size (*n* = 3). Our genetic statistics based on subsampling of the modern regions to the same sample size as the historical ones indicated that Randsfjorden was most affected by sample size—displayed by the largest standard deviations (Table S5).

Future studies, focusing on temporal genetic changes, should map available historical specimens in natural history collection prior to collecting modern data. In this way, one could allow targeted and regular modern sampling in historically well‐covered sites, ensuring sufficient sample sizes and preferably enabling direct population comparisons. Our overall measures of modern diversity, which were based on regions, were comparable to those obtained by Kyrkjeeide et al. (2020), which were based on populations (i.e., *H*_E_ = 0.316 vs. *H*_E_ = 0.30, *H*_O_ = 0.296 vs. *H*_O_ = 0.27, and *F*_IS_ = 0.062 vs. *F*_IS_ = 0.10, respectively). This may not always be the case, however, especially in cases of strong subpopulation structuring. Hence, the approach used herein may not be applicable for other species or certain areas of their distribution.

#### 4.2.3 Unrealized potential distribution?

We applied environmental niche modeling (ENM) to identify areas potentially suitable for northern dragonhead in Norway. Our ENM results suggest that the distribution of northern dragonhead is anchored in warmer and drier regions within Norway, more specifically areas with mean summer temperatures higher than 10°C and <~800 mm of mean annual precipitation (Figure 7b). These findings are in line with the early assumption by Sterner (1922) that the distribution of northern dragonhead is limited by low summer temperatures. In its current southeastern distribution in Norway, northern dragonhead is further restricted to areas of dry, calcareous meadows or steep, rough land like ledges along roads, in addition to extensively managed agricultural lands (Fægri & Danielsen, 1996). Further, east of its present distribution, the valleys are dominated by noncalcareous soils and bedrock not suitable for northern dragonhead (Fægri & Danielsen, 1996). However, our ENM results suggest areas representing potentially suitable climatic niche space for northern dragonhead in Trøndelag (central Norway), the inner parts of the fjords in the western part of the country, and in northeastern Norway (Figure 7c, deep red). The latter area may seem unlikely given the cold and long winters above the Arctic circle at approximately 70 degrees north. Notably, this area was suggested as suitable also for *Carex jemtlandica* (see Nygaard et al., 2021), which also has a mainly southeastern distribution in Norway. A number of vascular plant species of similar habitats to northern dragonhead display the same subcontinental distribution in Norway (e.g., A*rtemisia campestris*, *Brachypodium pinnatum*, *Carlina vulgaris*, *Crepis praemorsa*, *Draba nemorosa*, *Fragaria viridis*, *Tragopogon pratensis*, and *Veronica verna*; Fægri & Danielsen, 1996). Another group has extended beyond the mountains framing the southeastern lowlands and reached Central Norway or the inner, warm western fjords during the postglacial warm period (e.g., *Androsace septentrionalis*, *Calamagrostis arundinacea*, *Filipendula vulgaris*, *Myosotis ramosissima*, *M. stricta*, *Polygala amarella*, *Ranunculus polyanthemos*, *Saxifraga tridactylites*, *Sedum rupestre*, and *Viola collina*; Fægri & Danielsen, 1996). Northern dragonhead does indeed appear to vary in its habitat preference throughout its European distribution, occurring at rather high elevation in some areas (see Norwegian Directorate for Nature Management, 2010, and references therein). In *Flora Helvetica* (2018), northern dragonhead is reported as a subalpine species, in Switzerland reaching ca. 2000 m a.s.l. (similar to ca. 700 m a.s.l. in Norway). GBIF includes a record of northern dragonhead from as high elevation as 2365 m a.s.l. in Switzerland (gbif.org/occurrence/1851584929). It is therefore possible that dry and calcareous habitats in Finnmark in northeastern Norway may represent a suitable area for the species, to which it has so far not reached. This would imply that similar habitats in northern Finland, northern Sweden, and northwestern Russia could represent suitable areas for northern dragonhead survival, areas where the species does not occur today.

## 5 CONCLUSION

With this study, we demonstrate that, with the appropriate design procedures, the microfluidic SNP array technology is promising for genotyping old herbarium specimens; an invaluable source of information from the past. As expected, the SNP array picked up less genetic variability in the extra‐Norwegian specimens, likely due to both genetic divergence and the fact that the array was developed based on modern Norwegian samples alone. Our temporal genomic analyses of northern dragonhead in Norway show no signs of any severe reduction in population size in any of the studied regions. This may seem like good news, which indeed it might be if it is so that the populations have remained large enough to withstand the effect of genetic drift and inbreeding. The same results may, however, be due to a time lag in the response caused by the relatively long generation time of northern dragonhead. It is tempting to speculate whether our results could also be reflecting the ongoing climate change; increasing temperatures and less precipitation could potentially lead to an increase in connectivity and gene flow between neighboring populations and an expansion of the limits of currently suitable habitats. Regardless, the regional areas studied are genetically divergent across space, both from each other and clearly so from populations outside of Norway, rendering continued protection of the species and its regional genetic variation in Norway relevant. Our ENM results suggest that northern dragonhead has not yet reached its potential distribution in Norway. With the future inclusion of additional parameters (e.g., pH), ENM should prove useful for guiding management authorities in translocation for conservation initiatives.

## AUTHOR CONTRIBUTIONS

**Malene Nygaard:** Data curation (equal); formal analysis (lead); investigation (equal); methodology (equal); visualization (lead); writing – original draft (lead); writing – review and editing (equal). **Alexander Kopatz:** Formal analysis (supporting); investigation (equal); methodology (equal); supervision (supporting); visualization (supporting); writing – review and editing (supporting). **James D. M. Speed:** Formal analysis (supporting); investigation (equal); methodology (equal); visualization (supporting); writing – review and editing (supporting). **Michael D. Martin:** Conceptualization (equal); funding acquisition (equal); investigation (supporting); visualization (supporting); writing – review and editing (supporting). **Tommy Prestø:** Conceptualization (equal); data curation (supporting); funding acquisition (equal); writing – review and editing (supporting). **Oddmund Kleven:** Data curation (equal); investigation (equal); methodology (equal); supervision (equal); visualization (supporting); writing – review and editing (supporting). **Mika Bendiksby:** Conceptualization (equal); data curation (equal); funding acquisition (equal); investigation (equal); methodology (equal); project administration (lead); supervision (equal); visualization (supporting); writing – original draft (supporting); writing – review and editing (equal).

## CONFLICT OF INTEREST

The authors have no conflict of interest and no relevant financial or nonfinancial interests to disclose.

## Supporting information


Figure S1–S14

Table S1–S5

## Data Availability

The herbarium specimens used for the newly generated data are in the following public collections: LECB, O, TRH, and UPS, and voucher information provided in Table S1. The species occurrence records used herein are available from GBIF (see the link provided in chapter 2.4), and Tables S2 and S3 list the GBIF‐IDs lacking coordinates (i.e., our approximations based on specimen metadata) and those that were considered spurious, respectively. Both our newly produced SNP data and those produced by Kyrkjeeide et al. (2020) are available from DRYAD (https://doi.org/10.5061/dryad.c59zw3r8g).
