# Supplementary material for: Spatiotemporal monitoring of the rare northern dragonhead (Dracocephalum ruyschiana, Lamiaceae) — SNP genotyping and environmental niche modeling herbarium specimens
Source: Ecol Evol. 2022 Aug 12;12(8):e9187. doi: 10.1002/ece3.9187 (PMC9374565; doi:10.1002/ece3.9187)
Supplement: Supplementary file 1 — Figure S1–S14 [file ECE3-12-e9187-s001.docx]

**Species monitoring through space and time — combining microfluidic SNP genotyping and environmental niche modelling on herbarium specimens of the rare Northern dragonhead, *Dracocephalum ruyschiana* (Lamiaceae)**

**Supplementary Figures S1-S14**

**Supplementary Figures**

**
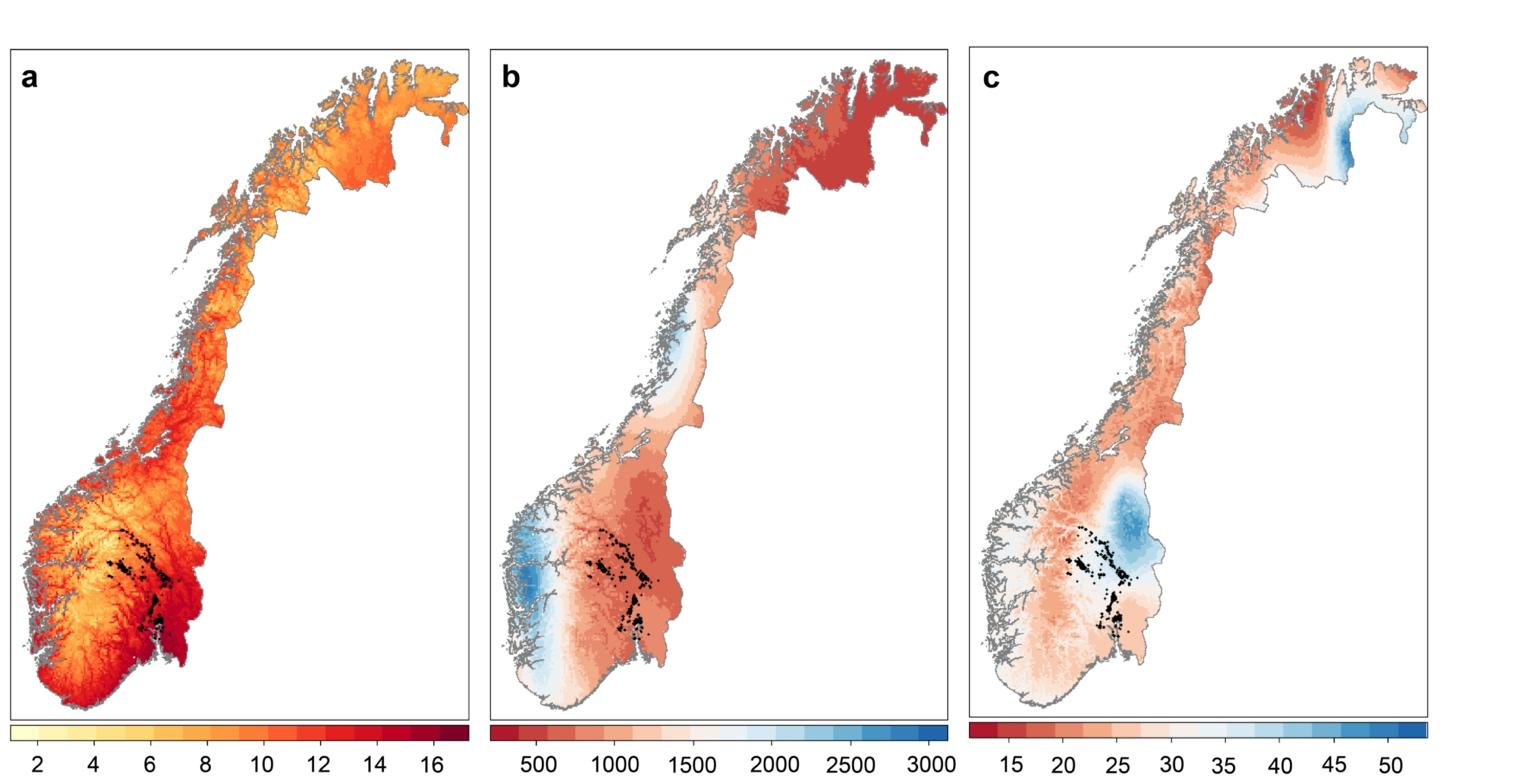
**

**Figure S1.** The selected climatic variables, used for environmental niche modeling, plotted across our study region (Norway) at 1-km resolution: **a)** mean summer temperature (^०^C), **b)** mean annual precipitation (mm), and **c)** precipitation seasonality (coefficient of variance of monthly precipitation). The spatial distribution of 4092 occurrence records of *Dracocephalum ruyschiana* is displayed with black dots.


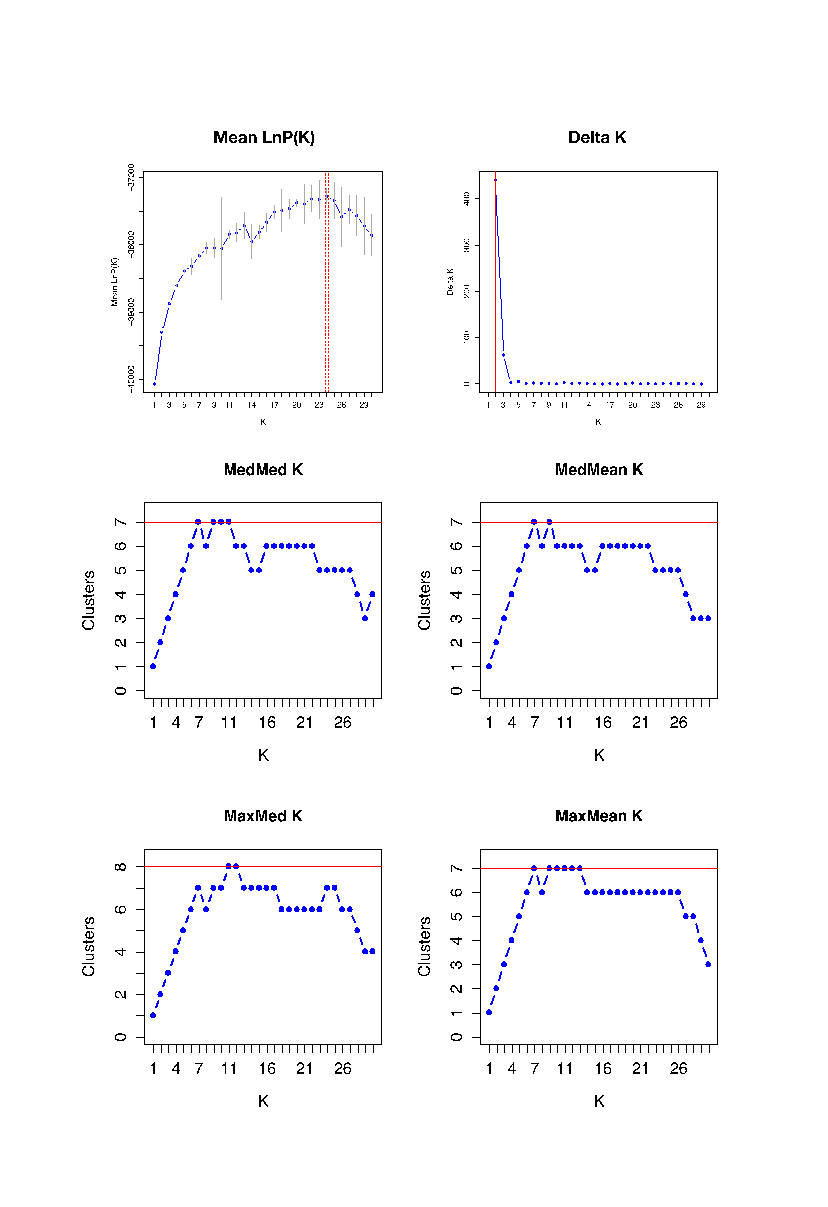


**Figure S2.** Cluster identification within 76 historical and 355 modern samples of Norwegian *D. ruyschiana* (NOR) based on output from Structure analysis using 92 SNPs. The optimal number of clusters are indicated by red lines for all optimization methods. Figures generated by StructureSelector (Li and Liu 2017).

**
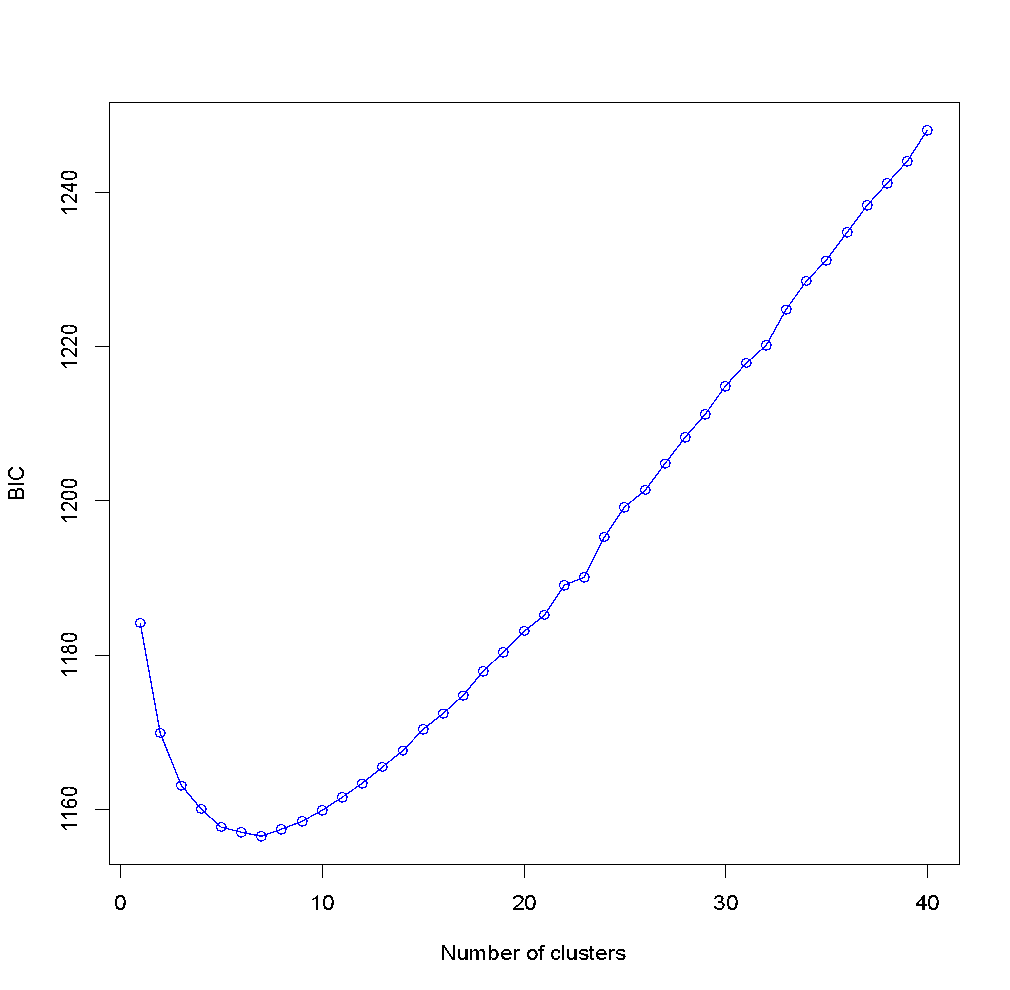
Figure S3.** Cluster identification using successive k-means within 76 historical and 355 modern samples of Norwegian *Dracocephalum ruyschiana* (NOR), produced by *find.cluster* in R environment. Optimal numbers of clusters yield lower BIC values.


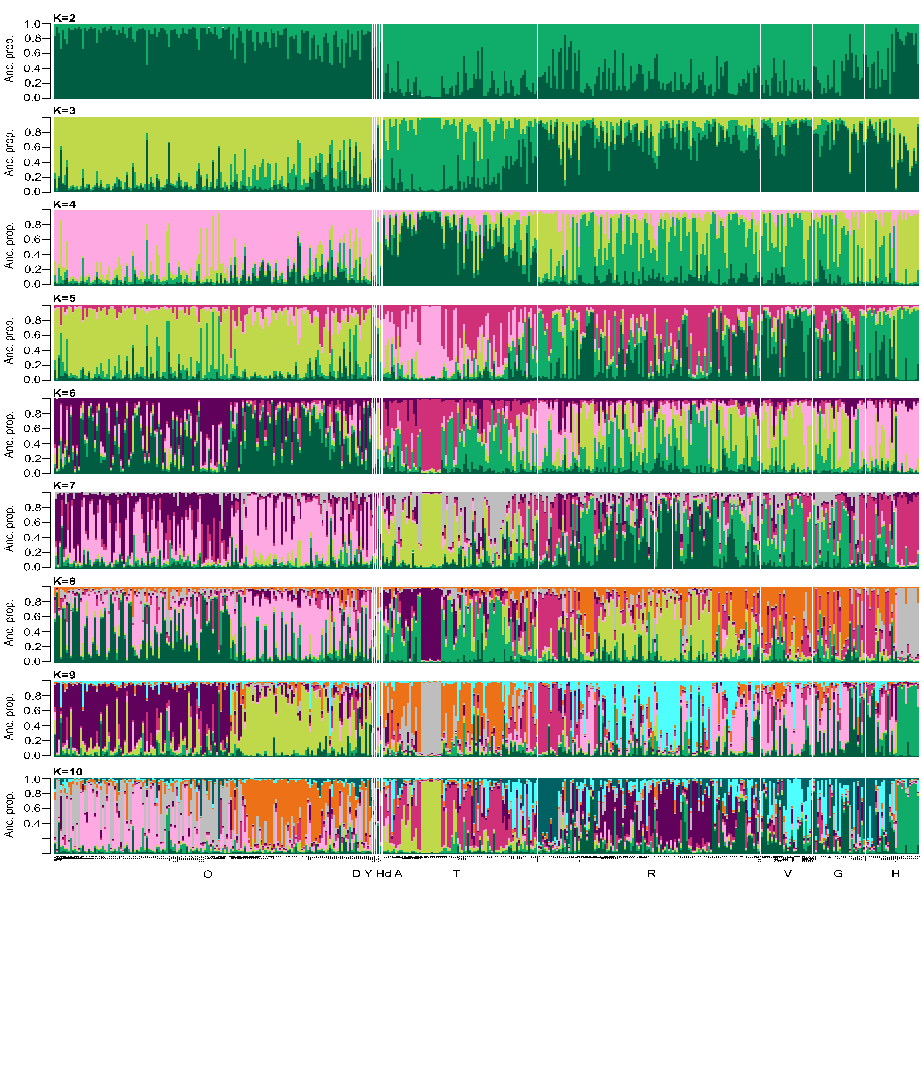


**Fig S4.** Structure results from *K*=2 to *K*=10 for our NOR dataset of *Dracocephalum ruyschiana*. Vertical bars represent individuals, and their ancestry proportion of each genetic cluster is displayed by the size of the colour segment. Samples are sorted by municipalities within the larger geographical regions, subsequently by modern and historical samples, respectively. Abbreviations: A=Agder, B=Buskerud, D=Drammensfjorden, G=Gudbrandsdalen, Hd=Hemsedal, O=Oslofjorden, Oy=Oslofjorden ytre, R=Randsfjorden, T=Tyrifjorden, V=Valdres.

**
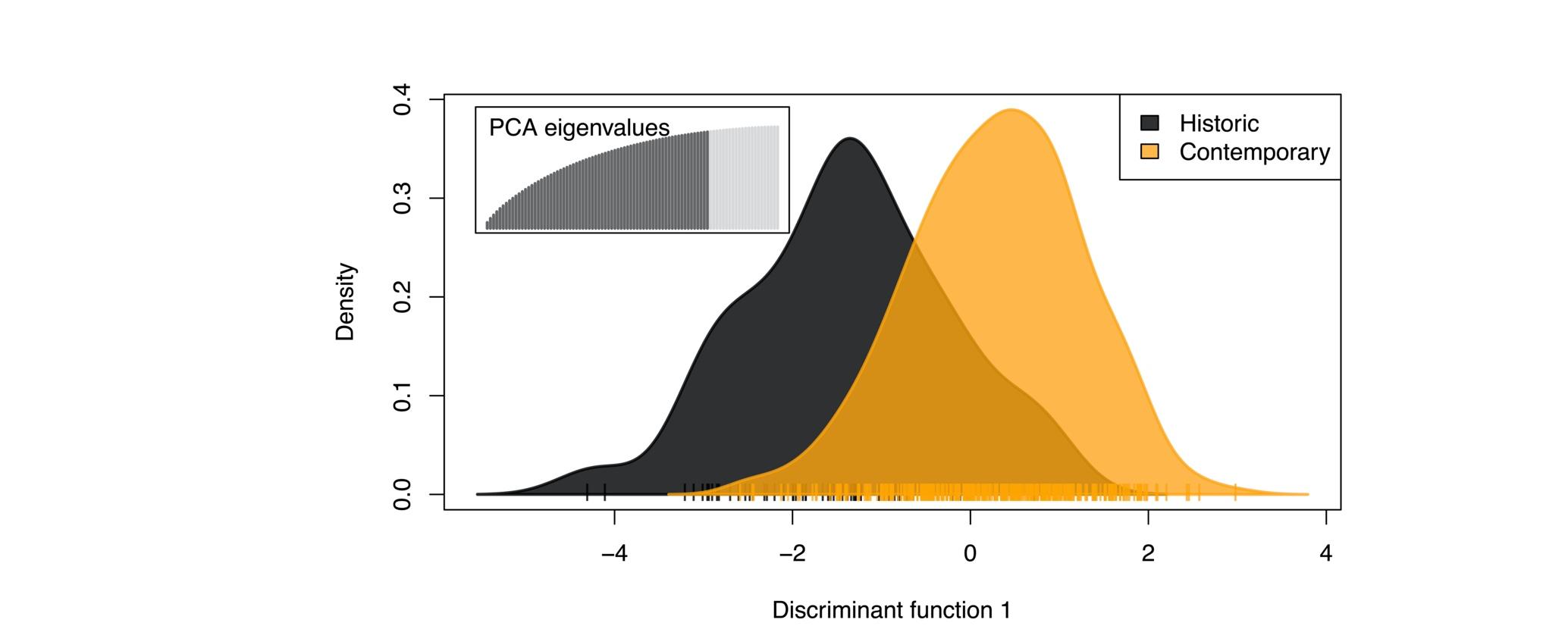
**

**Figure S5.** DAPC analysis for Norwegian *Dracocephalum ruyschiana* based on 92 SNPs. The density plot of each predefined group is presented by different colours: historical in black and modern in orange.

**
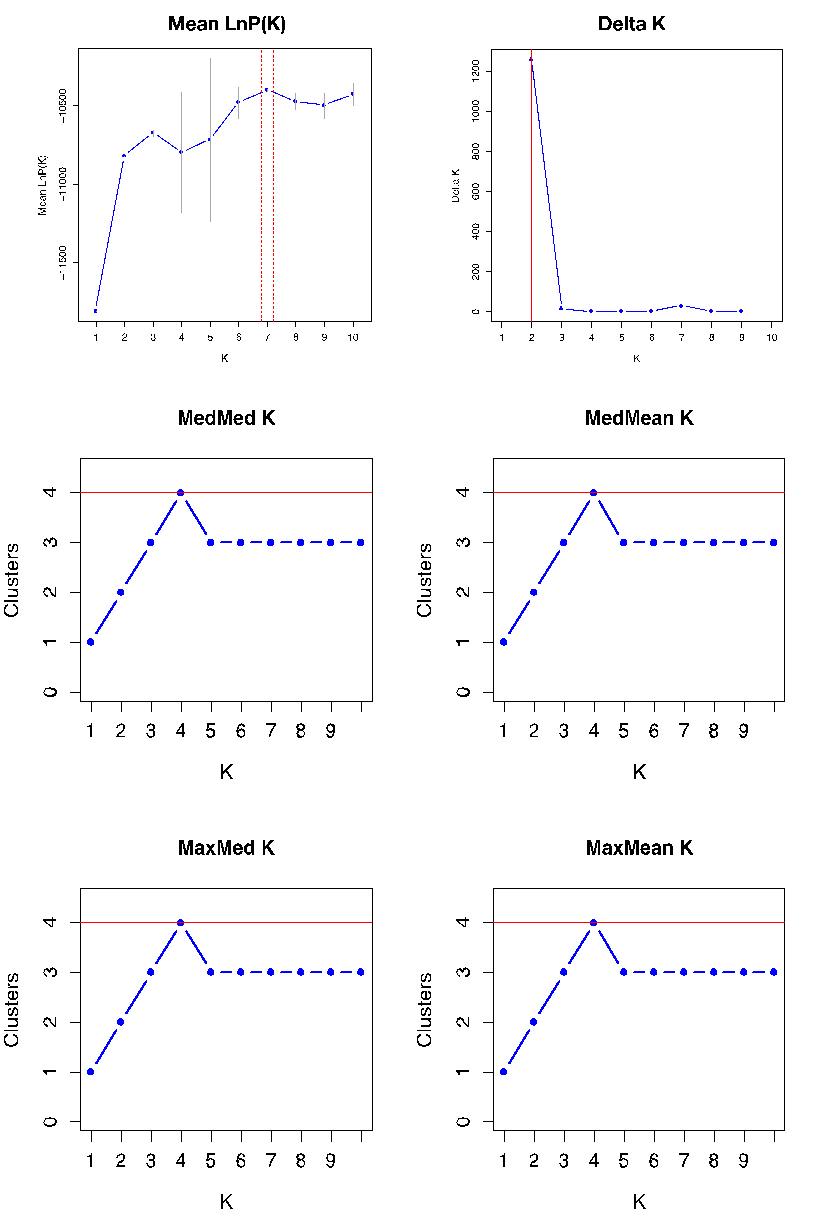
**

**Fig S6.** Cluster identification within 130 historical samples of European *D. ruyschiana* (GLOB) based on output from Structure analysis using 92 SNPs. The optimal number of clusters are indicated by red lines for all optimization methods. Figures generated by StructureSelector (Li and Liu 2017).

**
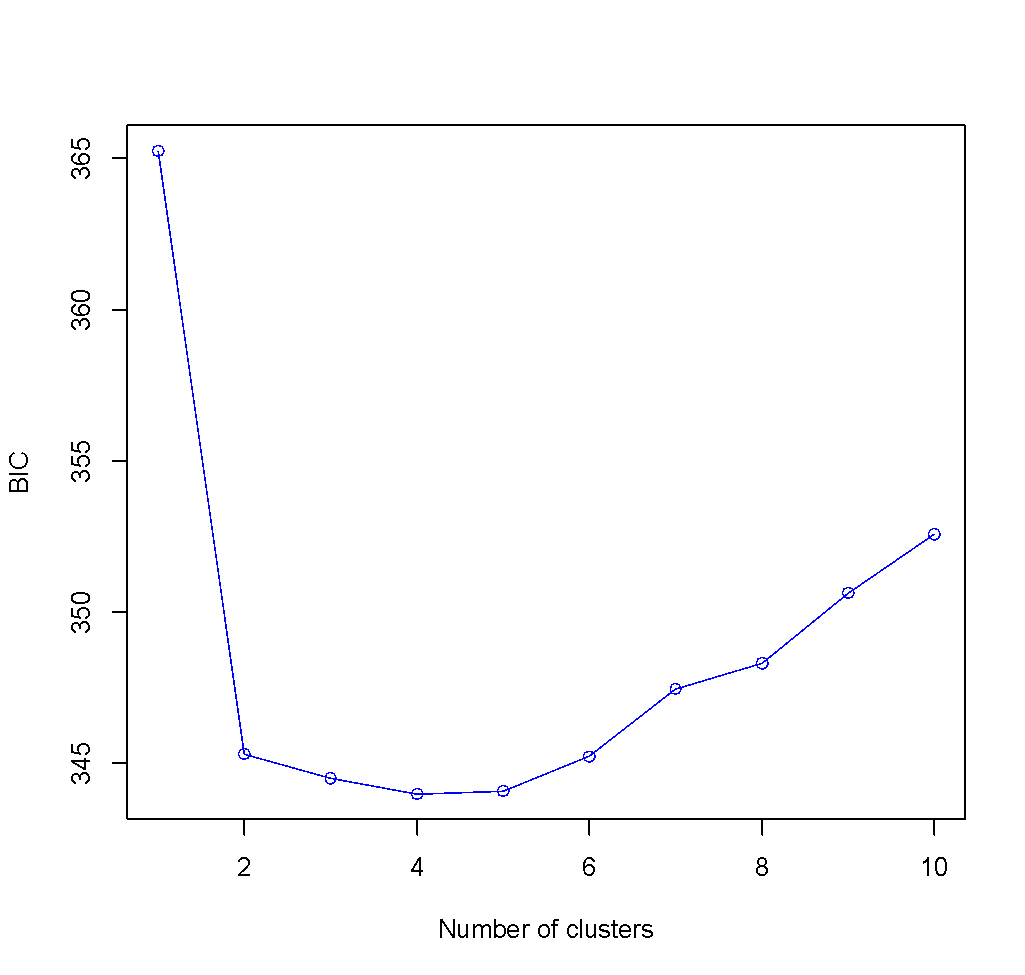
Fig S7.** Cluster identification using successive k-means within 130 historical samples of European *Dracocephalum ruyschiana* (GLOB), produced by *find.cluster* in R environment. Optimal numbers of clusters yield lower BIC values.


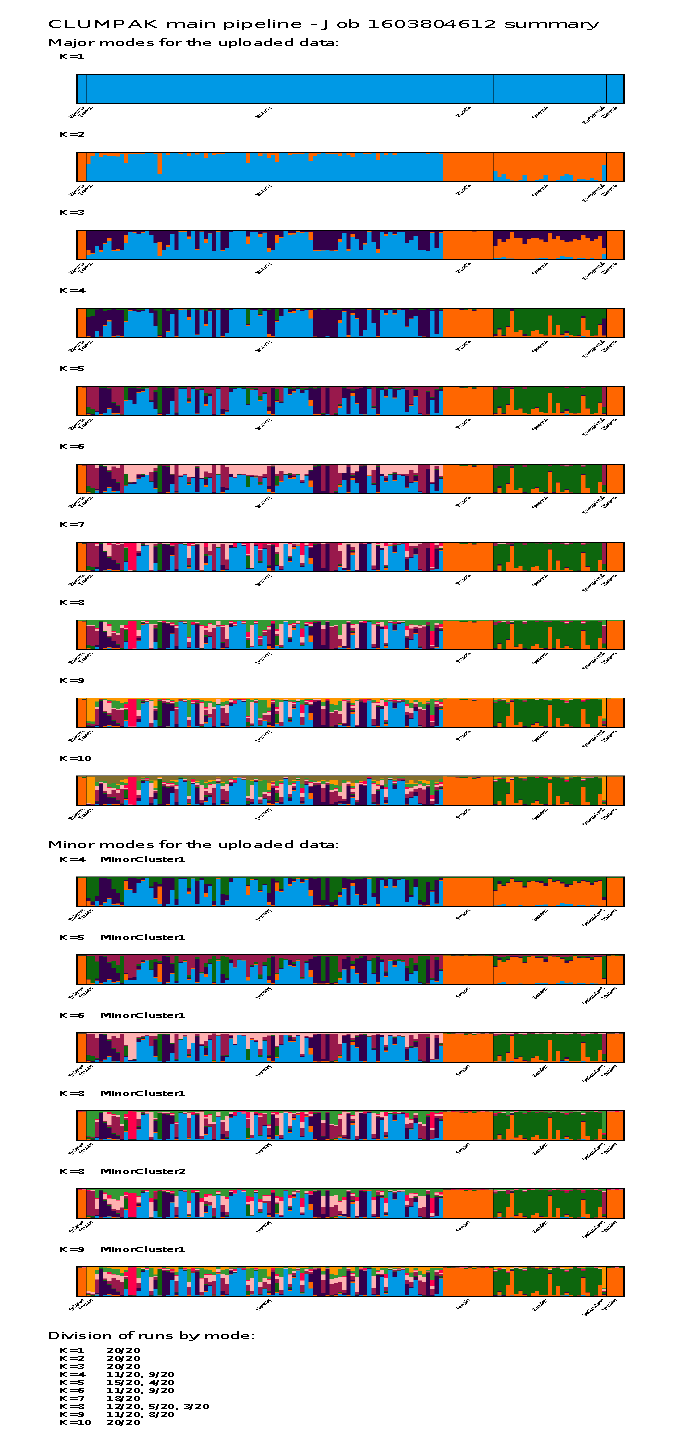


**Fig S8.** Structure results from *K*=2 to *K*=10 for GLOB. Vertical bars represent individuals, and their ancestry proportion of each genetic cluster is displayed by the size of the colour segment. Countries are separated by black vertical lines. Figur produced using the CULUMPAK server (http://clumpak.tau.ac.il/).

**
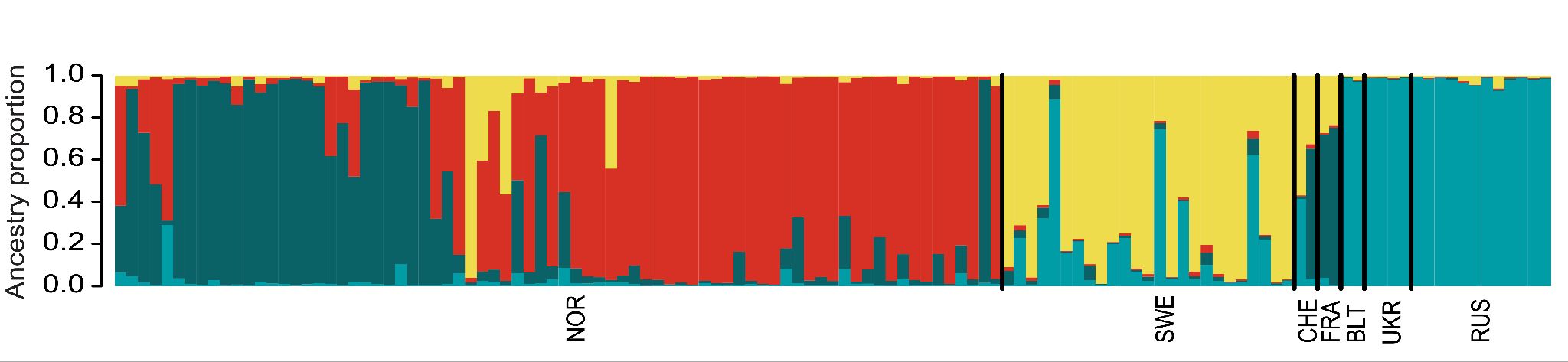
**

**Fig S9.** Structure results at optimal K=4 for the GLOB dataset of *Dracocephalum ruyschiana*, based on 92 SNPs. Vertical bars represent individuals, and their ancestry proportion of each genetic cluster is displayed by the size of the colour segment. The samples are sorted according to country of origin (separated by black vertical lines), and with increasing geographical distance from Norway. Abbreviations: BLT Belarus, CHE Switzerland , FRA France , NOR Norway, RUS Russia, SWE Sweden, and UKR Ukraine.

**
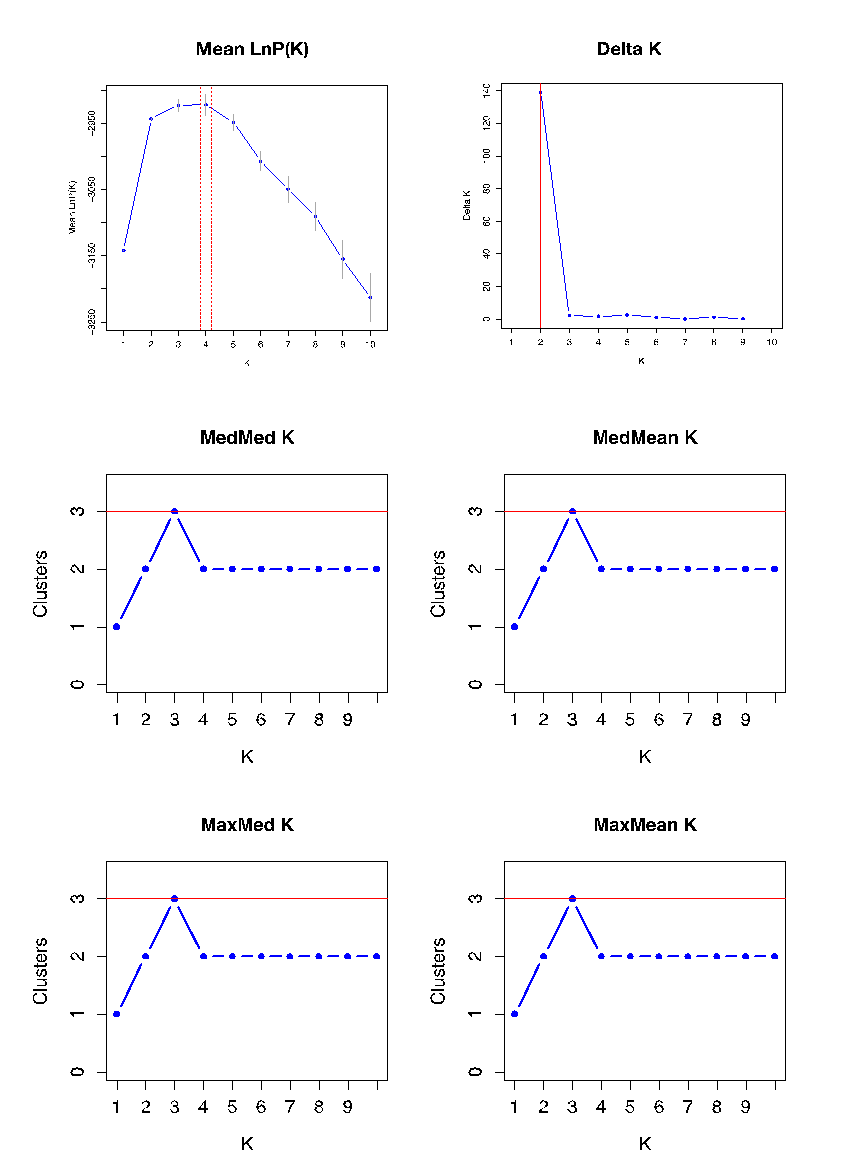
Fig S10.** Cluster identification within 47 historical samples of European *D. ruyschiana* (GLOB excluding Norway) based on output from Structure analysis using 92 SNPs. The optimal number of clusters are indicated by red lines for all optimization methods. Figures generated by StructureSelector (Li and Liu 2017).

**
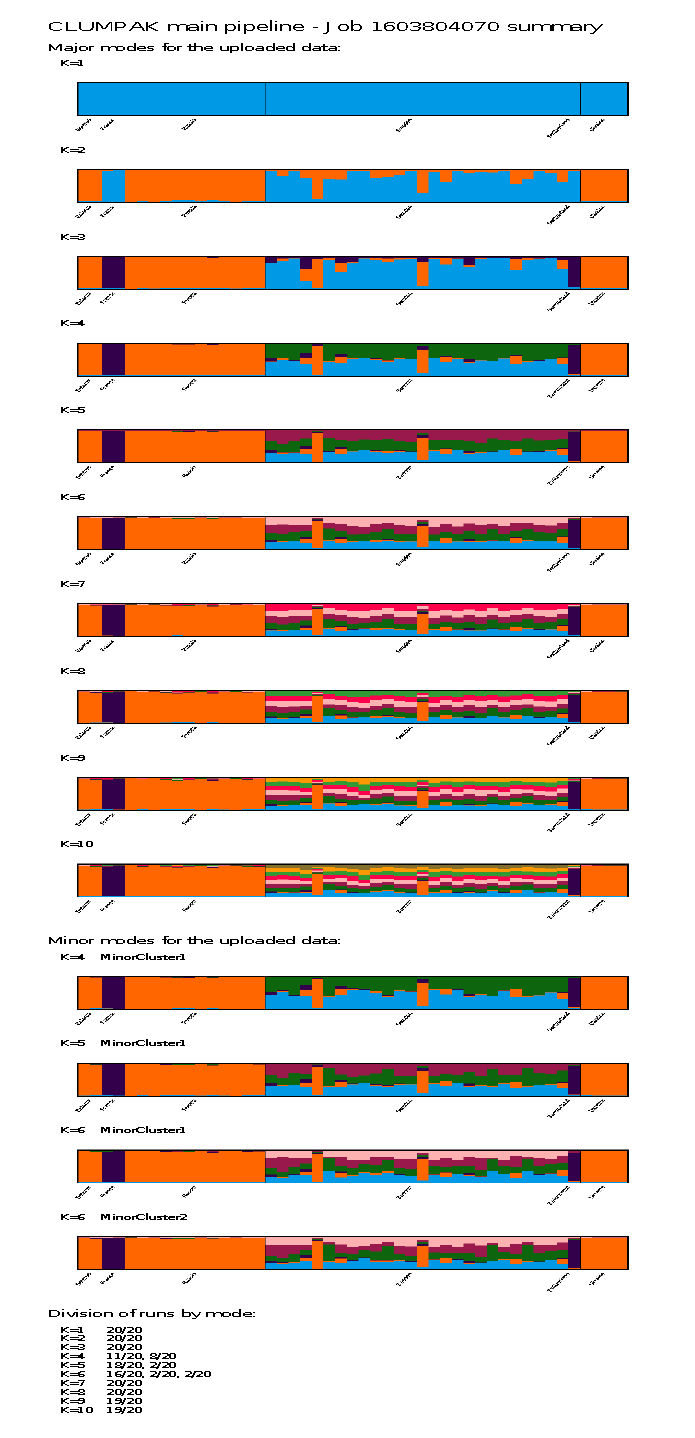
**

**Fig S11**. Structure results from *K*=2 to *K*=10 for GLOB, excluding all Norwegian samples. Vertical bars represent individuals, and their ancestry proportion of each genetic cluster is displayed by the size of the colour segment. Samples are sorted by municipalities within the larger geographical regions, subsequently by modern and historical samples, respectively. Figure produced using the CULUMPAK server (http://clumpak.tau.ac.il/).

**
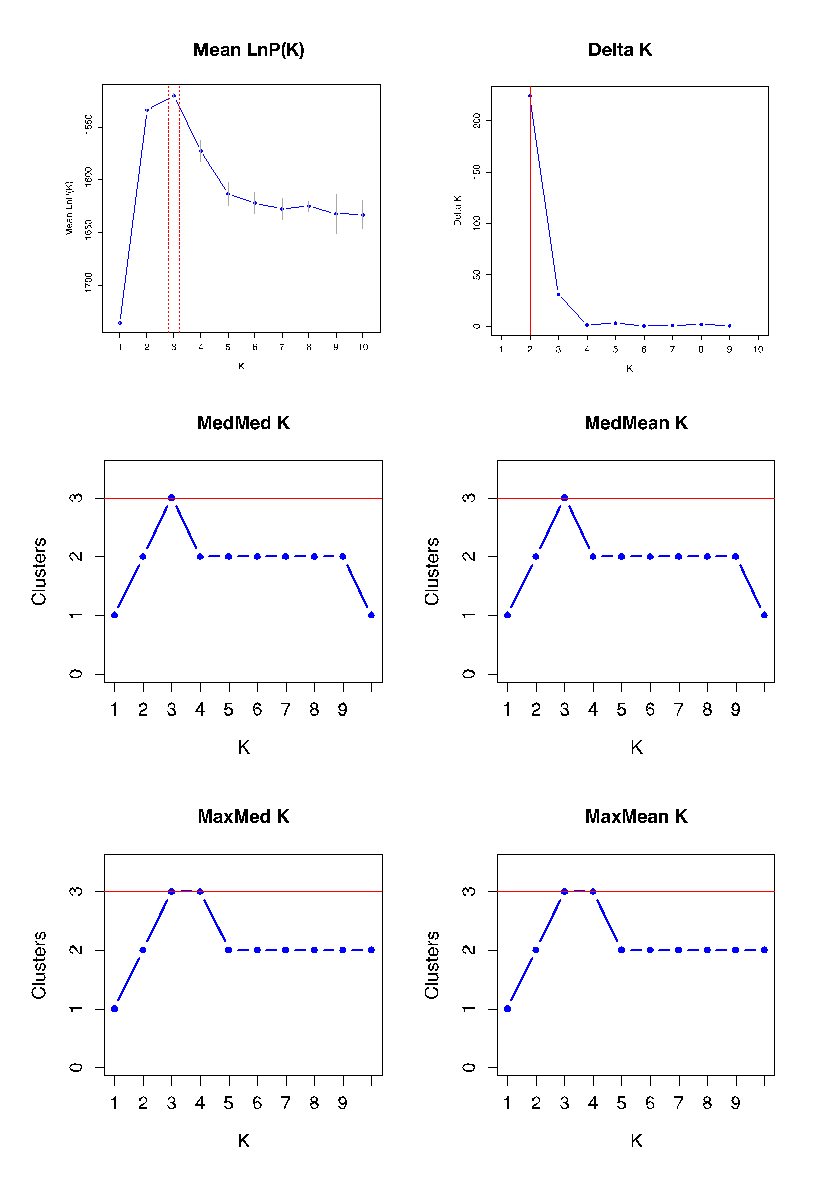
**

**Fig S12.** Cluster identification within 22 historical samples of European *D. ruyschiana* (GLOB reduced and balanced) based on output from Structure analysis using 92 SNPs. For countries with more than 4 samples we selected 4 random samples using the function sample() in R. The randomly selected samples include: 478352, 753564, 6526, 260883 from Norway, V-073875, V-038712, V-073850, 838063 from Sweden, and 2250131, V-069513, D-17, D-26 form Russia. The optimal number of clusters are indicated by red lines for all optimization methods. Figures generated by StructureSelector (Li and Liu 2017).


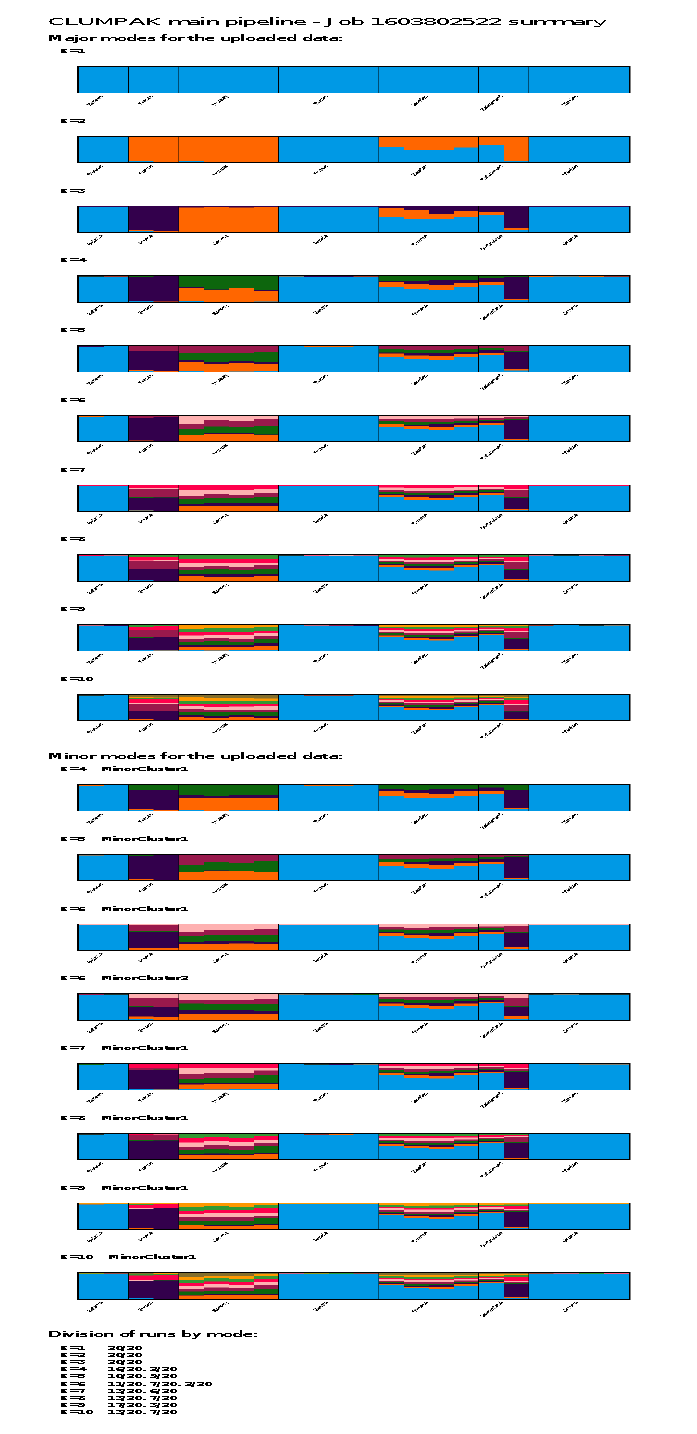


**Fig S13**. Structure results from *K*=2 to *K*=10 for GLOB, reduced and balanced. For countries with more than 4 samples we selected 4 random samples using the function sample() in R. The randomly selected samples include: 478352, 753564, 6526, 260883 from Norway, V-073875, V-038712, V-073850, 838063 from Sweden, and 2250131, V-069513, D-17, D-26 form Russia. Vertical bars represent individuals, and their ancestry proportion of each genetic cluster is displayed by the size of the colour segment. Samples are sorted by municipalities within the larger geographical regions, subsequently by modern and historical samples, respectively. Figure produced using the CULUMPAK server (<http://clumpak.tau.ac.il/>).


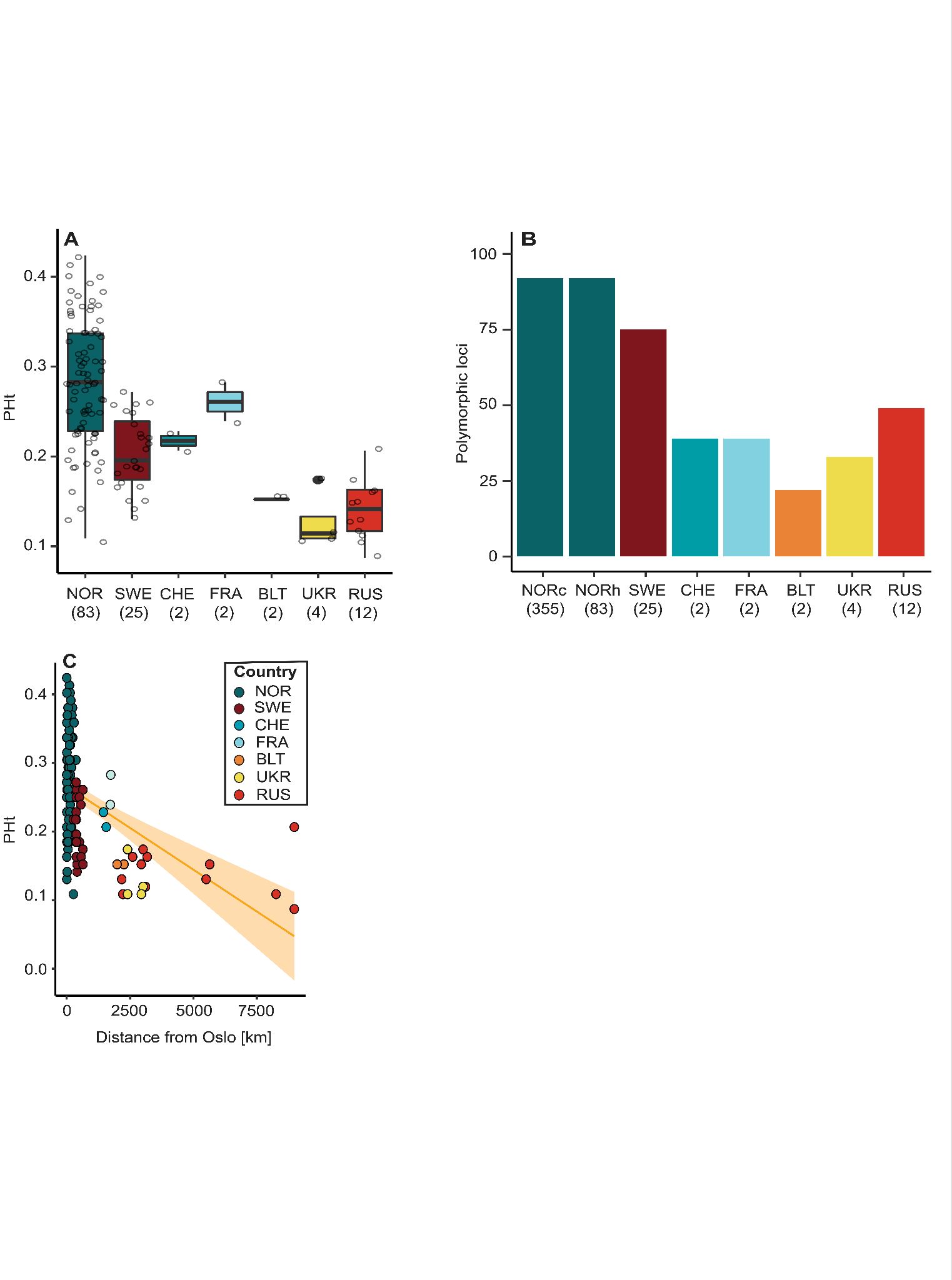


**Fig S14.** Display of various diversity measures across countries for our *Dracocephalum ruyschiana* SNP data (GLOB). The samples are sorted according to country of origin, and countries with increasing geographical distance from Norway. Abbreviations: BLT Belarus, CHE Switzerland , FRA France , NOR Norway, NORc Norway contemporary, NORh Norway historical, RUS Russia, SWE Sweden, and UKR Ukraine. **a)** Distribution of SNP genotyping diversity, measured as individual proportions of heterozygous loci (PHt), across 130 samples of *Dracocephalum ruyschiana*. The box hinges represent the first and third quartiles, the thick middle line median value, and the whiskers display the minimum and maximum value. Individual heterozygosity values are displayed as black points, and outliers as big, black dots. **b)** Number of polymorphic loci across countries for our *Dracocephalum ruyschiana* SNP data. **c)** Correlation between individual heterozygosity (PHt) and geographical distance from Norway (Oslo). Points represent separate samples, and their colour is the country of origin. The orange line is the regression line, and the orange zone is the 95% confidence interval.
