# Supplementary material for: Spatiotemporal monitoring of the rare northern dragonhead (Dracocephalum ruyschiana, Lamiaceae) — SNP genotyping and environmental niche modeling herbarium specimens
Source: Ecol Evol. 2022 Aug 12;12(8):e9187. doi: 10.1002/ece3.9187 (PMC9374565; doi:10.1002/ece3.9187)
Supplement: Supplementary file 2 — Table S1–S5 [file ECE3-12-e9187-s002.docx]

**Species monitoring through space and time — combining microfluidic SNP genotyping and environmental niche modelling on herbarium specimens of the rare Northern dragonhead, *Dracocephalum ruyschiana* (Lamiaceae)**

**Supplementary Tables S1-S5**

**Supplementary tables**

**Table S1.** List of all included herbarium specimens of *Dracocephalum ruyschiana* used for the molecular data production. In cases where specimen’s collection year was not specified, we used a conservative estimate based on available documentation (see Notes). DNA concentration was measured for 116 samples using Qubit, while mean DNA fragment length was approximated through gel electrophoresis (brightest band).

| **Sample ID** | **Herbarium Accronym** | **Accession number** | **Genus name** | **Species name** | **Country** | **Collection year** | **Latitude** | **Longitude** | **DNA concentration [ng/uL]** | **Mean DNA fragment length [bp]** | **Notes** |
| --- | --- | --- | --- | --- | --- | --- | --- | --- | --- | --- | --- |
| D-74 | O | 6511 | *Dracocephalum* | *ruyschiana* | Norway | 1939 | 60,8127 | 10,8474 | 20,6 | >1000 |  |
| D-75 | O | 6512 | *Dracocephalum* | *ruyschiana* | Norway | 1939 | 60,9271 | 10,6926 | 26,2 | 700 |  |
| D-76 | O | 6513 | *Dracocephalum* | *ruyschiana* | Norway | 1942 | 60,8977 | 10,8754 | 27,9 | 600 |  |
| D-77 | O | 6520 | *Dracocephalum* | *ruyschiana* | Norway | 1874 | 60,62647 | 11,34552 | 3,21 | 150 |  |
| D-78 | O | 6521 | *Dracocephalum* | *ruyschiana* | Norway | 1878 | 60,792 | 11,0391 | 3,31 | Not visable |  |
| D-79 | O | 6522 | *Dracocephalum* | *ruyschiana* | Norway | 1897 | 60,7914 | 11,0758 | 23,8 | 400 |  |
| D-80 | O | 6524 | *Dracocephalum* | *ruyschiana* | Norway | 1939 | 60,9263 | 10,8208 | 19,7 | 200 |  |
| D-81 | O | 6525 | *Dracocephalum* | *ruyschiana* | Norway | 1939 | 60,8004 | 11,1353 | 40,1 | 500 |  |
| D-82 | O | 6526 | *Dracocephalum* | *ruyschiana* | Norway | 1896 | 61,3059 | 11,4552 | 1,26 | Not visable |  |
| VM12 | TRH | 48438 | *Dracocephalum* | *ruyschiana* | Norway | 2008 | 61,16884 | 8,43427 | No data | No data |  |
| D-51 | O | 120988 | *Dracocephalum* | *ruyschiana* | Norway | 1909 | 59,8951 | 10,7319 | 14 | 250 |  |
| D-120 | O | 146561 | *Dracocephalum* | *ruyschiana* | Norway | 1880 | 58,1469 | 7,9977 | 7,62 | 150 |  |
| D-83 | O | 156500 | *Dracocephalum* | *ruyschiana* | Norway | 1890 | 61,8758 | 9,1502 | 9,06 | 150 |  |
| VM09 | TRH | 157645 | *Dracocephalum* | *ruyschiana* | Norway | 1996 | 59,83952 | 10,50773 | No data | No data |  |
| VM02 | TRH | 177634 | *Dracocephalum* | *ruyschiana* | Norway | 1920 | 59,89514 | 10,73191 | No data | No data |  |
| VM13 | TRH | 209441 | *Dracocephalum* | *ruyschiana* | Norway | 1907 | 61,10609 | 10,47489 | No data | No data |  |
| VM15 | TRH | 217018 | *Dracocephalum* | *ruyschiana* | Norway | 1924 | 61,32357 | 10,2796 | No data | No data |  |
| VM16 | TRH | 217020 | *Dracocephalum* | *ruyschiana* | Norway | 1905 | 60,094 | 10,2137 | No data | No data | pre 1905 |
| VM03 | TRH | 217024 | *Dracocephalum* | *ruyschiana* | Norway | 1961 | 59,89514 | 10,73191 | No data | No data |  |
| VM04 | TRH | 217025 | *Dracocephalum* | *ruyschiana* | Norway | 1915 | 59,88106 | 10,77763 | No data | No data |  |
| VM05 | TRH | 217031 | *Dracocephalum* | *ruyschiana* | Norway | 1913 | 59,86787 | 10,75639 | No data | No data |  |
| VM10 | TRH | 217036 | *Dracocephalum* | *ruyschiana* | Norway | 1903 | 60,80369 | 11,09969 | No data | No data |  |
| VM11 | TRH | 248089 | *Dracocephalum* | *ruyschiana* | Norway | 1949 | 59,71832 | 10,04001 | No data | No data |  |
| VM01 | TRH | 249028 | *Dracocephalum* | *ruyschiana* | Norway | 1850 | 59,9085 | 10,7433 | No data | No data | pre 1850 |
| D-112 | O | 260879 | *Dracocephalum* | *ruyschiana* | Norway | 1907 | 60,8855 | 8,4777 | 27,5 | 450 |  |
| D-113 | O | 260883 | *Dracocephalum* | *ruyschiana* | Norway | 1904 | 60,1248 | 10,2778 | 37,9 | 700 |  |
| D-114 | O | 260886 | *Dracocephalum* | *ruyschiana* | Norway | 1915 | 59,5245 | 10,4494 | 34,6 | 1000 |  |
| D-115 | O | 260889 | *Dracocephalum* | *ruyschiana* | Norway | 1904 | 60,0799 | 10,276 | 32 | 700 |  |
| D-116 | O | 260890 | *Dracocephalum* | *ruyschiana* | Norway | 1901 | 60,05548 | 10,29156 | 15,3 | 300 |  |
| D-117 | O | 260899 | *Dracocephalum* | *ruyschiana* | Norway | 1968 | 60,05548 | 10,29156 | 28,5 | >1000 |  |
| D-118 | O | 260904 | *Dracocephalum* | *ruyschiana* | Norway | 1895 | 60,0105 | 9,9954 | 6,6 | 200 |  |
| VM14 | TRH | 300918 | *Dracocephalum* | *ruyschiana* | Norway | 1985 | 61,83911 | 9,2928 | No data | No data |  |
| VM06 | TRH | 305054 | *Dracocephalum* | *ruyschiana* | Norway | 1969 | 59,8615 | 10,75724 | No data | No data |  |
| D-121 | O | 315558 | *Dracocephalum* | *ruyschiana* | Sweden | 1997 | 58,263 | 13,755 | 27,6 | >1000 |  |
| D-52 | O | 422161 | *Dracocephalum* | *ruyschiana* | Norway | 1844 | 59,5981 | 11,0816 | 24,2 | 300 |  |
| D-53 | O | 478295 | *Dracocephalum* | *ruyschiana* | Norway | 1926 | 59,9138 | 10,681 | 17,7 | >1000 |  |
| D-54 | O | 478299 | *Dracocephalum* | *ruyschiana* | Norway | 1960 | 59,9084 | 10,6816 | 7,48 | 150 |  |
| D-55 | O | 478300 | *Dracocephalum* | *ruyschiana* | Norway | 1892 | 59,9445 | 10,7363 | 22,5 | 300 |  |
| D-56 | O | 478301 | *Dracocephalum* | *ruyschiana* | Norway | 1883 | 59,8857 | 10,7689 | 19,5 | 350 |  |
| D-57 | O | 478302 | *Dracocephalum* | *ruyschiana* | Norway | 1854 | 59,8811 | 10,7776 | 3,79 | 100 |  |
| D-58 | O | 478307 | *Dracocephalum* | *ruyschiana* | Norway | 1905 | 59,8951 | 10,7319 | 5,17 | 150 |  |
| D-59 | O | 478313 | *Dracocephalum* | *ruyschiana* | Norway | 1896 | 59,9084 | 10,6816 | 4,34 | 200 |  |
| D-60 | O | 478323 | *Dracocephalum* | *ruyschiana* | Norway | 1913 | 59,9084 | 10,6816 | 12,5 | 350 |  |
| D-61 | O | 478326 | *Dracocephalum* | *ruyschiana* | Norway | 1910 | 59,8951 | 10,7319 | 17,9 | 250 |  |
| D-62 | O | 478328 | *Dracocephalum* | *ruyschiana* | Norway | 1910 | 59,8951 | 10,7319 | 17,9 | 450 |  |
| D-63 | O | 478329 | *Dracocephalum* | *ruyschiana* | Norway | 1947 | 59,9494 | 10,7007 | 29,2 | 250 |  |
| D-67 | O | 478338 | *Dracocephalum* | *ruyschiana* | Norway | 1904 | 59,8694 | 10,5261 | 40,9 | 700 |  |
| D-68 | O | 478343 | *Dracocephalum* | *ruyschiana* | Norway | 1862 | 59,8966 | 10,6176 | 9,52 | 150 | M.N. Blytt (1789-1862) |
| D-69 | O | 478344 | *Dracocephalum* | *ruyschiana* | Norway | 1890 | 59,8966 | 10,6176 | 7,55 | 200 |  |
| D-70 | O | 478347 | *Dracocephalum* | *ruyschiana* | Norway | 1896 | 59,8594 | 10,5399 | 8,69 | 200 |  |
| D-71 | O | 478350 | *Dracocephalum* | *ruyschiana* | Norway | 1937 | 59,8697 | 10,7225 | 26,5 | 1000 |  |
| D-72 | O | 478352 | *Dracocephalum* | *ruyschiana* | Norway | 1927 | 59,6886 | 10,7319 | 10,7 | 150 |  |
| D-73 | O | 478353 | *Dracocephalum* | *ruyschiana* | Norway | 1935 | 59,7641 | 10,7892 | 20,1 | 300 |  |
| D-84 | O | 701456 | *Dracocephalum* | *ruyschiana* | Norway | 1869 | 60,66907 | 9,65721 | 17,8 | 250 |  |
| D-85 | O | 701459 | *Dracocephalum* | *ruyschiana* | Norway | 1868 | 60,66907 | 9,65721 | 10,6 | 200 |  |
| D-86 | O | 701460 | *Dracocephalum* | *ruyschiana* | Norway | 1891 | 61,56336 | 10,29904 | 14,3 | 200 |  |
| D-87 | O | 701462 | *Dracocephalum* | *ruyschiana* | Norway | 1898 | 61,56336 | 10,29904 | 7,06 | 150 |  |
| D-88 | O | 701466 | *Dracocephalum* | *ruyschiana* | Norway | 1898 | 61,56336 | 10,29904 | 25,3 | 250 |  |
| D-89 | O | 701468 | *Dracocephalum* | *ruyschiana* | Norway | 1923 | 61,2762 | 10,1602 | 22,7 | 300 |  |
| D-90 | O | 701470 | *Dracocephalum* | *ruyschiana* | Norway | 1895 | 61,13415 | 10,39176 | 26,2 | 400 |  |
| D-92 | O | 701474 | *Dracocephalum* | *ruyschiana* | Norway | 1896 | 61,25685 | 9,09393 | 24 | 250 | N. Aars-Nicolaysen (1832-1896) |
| D-93 | O | 701477 | *Dracocephalum* | *ruyschiana* | Norway | 1885 | 60,28641 | 10,38658 | 4,89 | 150 |  |
| D-94 | O | 753524 | *Dracocephalum* | *ruyschiana* | Norway | 1906 | 61,1467 | 8,5839 | 18 | >1000 |  |
| D-95 | O | 753525 | *Dracocephalum* | *ruyschiana* | Norway | 1875 | 61,05606 | 8,90577 | 35,7 | 500 |  |
| D-96 | O | 753530 | *Dracocephalum* | *ruyschiana* | Norway | 1907 | 60,3595 | 10,5861 | 21,3 | 300 |  |
| D-97 | O | 753536 | *Dracocephalum* | *ruyschiana* | Norway | 1906 | 61,13415 | 10,39176 | 20,5 | 900 |  |
| D-98 | O | 753541 | *Dracocephalum* | *ruyschiana* | Norway | 1909 | 60,9882 | 9,2403 | 23,4 | 900 |  |
| D-99 | O | 753543 | *Dracocephalum* | *ruyschiana* | Norway | 1920 | 60,8485 | 10,0802 | 13,8 | 500 |  |
| D-100 | O | 753546 | *Dracocephalum* | *ruyschiana* | Norway | 1909 | 60,8546 | 9,9939 | 31,8 | 800 |  |
| D-101 | O | 753550 | *Dracocephalum* | *ruyschiana* | Norway | 1920 | 60,43395 | 10,53107 | 9,58 | 200 |  |
| D-102 | O | 753552 | *Dracocephalum* | *ruyschiana* | Norway | 1870 | 60,66907 | 9,65721 | 23,2 | 300 |  |
| D-103 | O | 753554 | *Dracocephalum* | *ruyschiana* | Norway | 1874 | 60,66907 | 9,65721 | 28,8 | 500 |  |
| D-104 | O | 753555 | *Dracocephalum* | *ruyschiana* | Norway | 1938 | 60,66907 | 9,65721 | 8,44 | 200 | Eugen Jørgensen (1862-1938) |
| D-105 | O | 753564 | *Dracocephalum* | *ruyschiana* | Norway | 1903 | 61,2217 | 10,4496 | 26,5 | 550 |  |
| D-106 | O | 753567 | *Dracocephalum* | *ruyschiana* | Norway | 1897 | 61,3336 | 10,3155 | 32 | >1000 |  |
| D-107 | O | 753568 | *Dracocephalum* | *ruyschiana* | Norway | 1894 | 61,2217 | 10,4496 | 31,3 | 1000 |  |
| D-108 | O | 753569 | *Dracocephalum* | *ruyschiana* | Norway | 1892 | 61,32771 | 10,51106 | 20,9 | 450 |  |
| D-109 | O | 753571 | *Dracocephalum* | *ruyschiana* | Norway | 1930 | 61,25685 | 9,09393 | 21,6 | 600 |  |
| D-110 | O | 753572 | *Dracocephalum* | *ruyschiana* | Norway | 1892 | 61,0612 | 9,0343 | 20,9 | 550 | M.N. Blytt (1789-1862), N.G. Moe (1812–1892) |
| D-111 | O | 753573 | *Dracocephalum* | *ruyschiana* | Norway | 1892 | 61,25685 | 9,09393 | 15,6 | 300 | M.N. Blytt (1789-1862), N.G. Moe (1812–1892) |
| D-119 | O | 753574 | *Dracocephalum* | *ruyschiana* | Norway | 1879 | 59,3795 | 10,526 | 10,8 | 200 |  |
| D-64 | O | 753577 | *Dracocephalum* | *ruyschiana* | Norway | 1820 | 59,97258 | 10,72237 | 16,8 | 100 |  |
| D-65 | O | 753579 | *Dracocephalum* | *ruyschiana* | Norway | 1834 | 59,97258 | 10,72237 | 10,5 | 150 |  |
| D-66 | O | 753585 | *Dracocephalum* | *ruyschiana* | Norway | 1826 | 59,8811 | 10,7776 | 14,9 | 250 |  |
| D-122 | O | 838060 | *Dracocephalum* | *ruyschiana* | Sweden | 1918 | 58,8125 | 14,254167 | 1,94 | 150 |  |
| D-123 | O | 838061 | *Dracocephalum* | *ruyschiana* | Sweden | 1882 | 56,876944 | 14,809167 | 12,3 | 200 |  |
| D-124 | O | 838062 | *Dracocephalum* | *ruyschiana* | Sweden | 1854 | 58,25 | 12,416667 | 12,7 | 150 |  |
| D-125 | O | 838063 | *Dracocephalum* | *ruyschiana* | Sweden | 1893 | 57,35 | 14,466667 | 20,6 | 600 |  |
| D-126 | O | 838064 | *Dracocephalum* | *ruyschiana* | Sweden | 1893 | 57,35 | 14,466667 | 25,1 | 500 |  |
| D-127 | O | 838065 | *Dracocephalum* | *ruyschiana* | Sweden | 1861 | na | na | 23,7 | 350 |  |
| D-129 | O | 838068 | *Dracocephalum* | *ruyschiana* | Russia | 1910 | 61,7 | 30,666667 | 29,8 | 900 |  |
| D-130 | O | 2250125 | *Dracocephalum* | *ruyschiana* | France | na | 44,92 | 6,36 | 4,85 | 150 |  |
| D-131 | O | 2250126 | *Dracocephalum* | *ruyschiana* | Switzerland | 1917 | 46,7 | 10,1 | 19,7 | >1000 |  |
| D-133 | O | 2250128 | *Dracocephalum* | *ruyschiana* | Russia | 1914 | 53,72 | 91,47 | 3,22 | 200 |  |
| D-134 | O | 2250129 | *Dracocephalum* | *ruyschiana* | France | 1830 | 44,67 | 6,65 | 5,65 | 150 | pre 1830 |
| D-135 | O | 2250130 | *Dracocephalum* | *ruyschiana* | Switzerland | na | 46,25 | 7,02 | 6,64 | 150 |  |
| D-136 | O | 2250131 | *Dracocephalum* | *ruyschiana* | Russia | 1914 | 53,72 | 91,47 | 17,7 | 300 |  |
| D-40 | UPS | V-038712 | *Dracocephalum* | *ruyschiana* | Sweden | 1850 | 58,13 | 13,6 | 3,38 | 100 | Collection year: 1830-1850 |
| D-49 | UPS | V-069513 | *Dracocephalum* | *ruyschiana* | Russia | 1927 | 56,826 | 85,001 | 8,59 | 200 |  |
| D-31 | UPS | V-073846 | *Dracocephalum* | *ruyschiana* | Sweden | 1849 | 57,42 | 15,25 | 1,66 | 100 |  |
| D-32 | UPS | V-073847 | *Dracocephalum* | *ruyschiana* | Sweden | 1860 | 57,35 | 14,48 | 14,7 | 150 |  |
| D-47 | UPS | V-073850 | *Dracocephalum* | *ruyschiana* | Sweden | 1858 | 58,481 | 16,316 | 3,23 | 100 |  |
| D-43 | UPS | V-073859 | *Dracocephalum* | *ruyschiana* | Sweden | 1876 | 58,124 | 13,413 | 15,2 | 150 |  |
| D-38 | UPS | V-073860 | *Dracocephalum* | *ruyschiana* | Sweden | 1871 | 58,27 | 13,71 | 10,5 | 200 |  |
| D-39 | UPS | V-073863 | *Dracocephalum* | *ruyschiana* | Sweden | 1875 | 58,303 | 13,788 | 4,15 | 100 |  |
| D-41 | UPS | V-073867 | *Dracocephalum* | *ruyschiana* | Sweden | 1915 | 57,93 | 13,49 | 9,87 | 200 |  |
| D-45 | UPS | V-073873 | *Dracocephalum* | *ruyschiana* | Sweden | 1922 | 57,899 | 13,478 | 16,1 | 250 |  |
| D-35 | UPS | V-073874 | *Dracocephalum* | *ruyschiana* | Sweden | 1890 | 57,84 | 13,65 | 1,9 | 100 |  |
| D-44 | UPS | V-073875 | *Dracocephalum* | *ruyschiana* | Sweden | 1929 | 57,861 | 13,553 | 13,4 | 400 |  |
| D-36 | UPS | V-073880 | *Dracocephalum* | *ruyschiana* | Sweden | 1854 | 58,19 | 13,51 | 3,14 | 100 |  |
| D-42 | UPS | V-073883 | *Dracocephalum* | *ruyschiana* | Sweden | 1854 | 58,06 | 13,66 | 6,37 | 150 |  |
| D-37 | UPS | V-073888 | *Dracocephalum* | *ruyschiana* | Sweden | 1877 | 58,13 | 13,77 | 1,17 | 100 |  |
| D-50 | UPS | V-073892 | *Dracocephalum* | *ruyschiana* | Sweden | 1847 | 59,5813 | 16,5105 | 17,1 | 200 |  |
| D-48 | UPS | V-146841 | *Dracocephalum* | *ruyschiana* | Russia | 2003 | 62,0115 | 34,1358 | 27,6 | >1000 |  |
| D-34 | UPS | V-230864 | *Dracocephalum* | *ruyschiana* | Sweden | 1917 | 58,81 | 14,25 | 2,92 | 100 |  |
| D-46 | UPS | V-586196 | *Dracocephalum* | *ruyschiana* | Sweden | na | 58,481 | 16,316 | 11,6 | 200 |  |
| D-17 | LECB |  | *Dracocephalum* | *ruyschiana* | Russia | 1917 | 55,1644 | 61,4368 | 10,1 | 600 | I.M. Krasheninnikov 17.06.1916 |
| D-18 | LECB |  | *Dracocephalum* | *ruyschiana* | Russia | 1995 | 55,0152 | 60,1588 | 41,3 | >1000 | V.V. Byalt no:15 |
| D-19 | LECB |  | *Dracocephalum* | *ruyschiana* | Russia | 1866 | 59,9343 | 30,3351 | 2,96 | 200 | Flora Ingrieae 1866 |
| D-21 | LECB |  | *Dracocephalum* | *ruyschiana* | Belarus | 1862 | 53,9007 | 30,3314 | 3,07 | 200 | N. Downar 1862 |
| D-22 | LECB |  | *Dracocephalum* | *ruyschiana* | Belarus | 1990 | 54,2042 | 27,8532 | 5,33 | 350 | N.N. Tsvelev 17.10.1990 |
| D-23 | LECB |  | *Dracocephalum* | *ruyschiana* | Russia | 1894 | 54,8583 | 37,5541 | 5,76 | 300 | s.n. 09.05.1894 |
| D-24 | LECB |  | *Dracocephalum* | *ruyschiana* | Russia | 1976 | 54,9833 | 39,0333 | 12,3 | >1000 | V. Tikhomirov & N. Lavrova 02.07.1976 |
| D-25 | LECB |  | *Dracocephalum* | *ruyschiana* | Russia | 1898 | 52,745 | 36,485 | 4,02 | 200 | V.V. Adamov 01.06.1898 |
| D-26 | LECB |  | *Dracocephalum* | *ruyschiana* | Russia | 1970 | 51,1916 | 37,65 | 36,7 | >1000 | N.P. Litvinova 4924 |
| D-27 | LECB |  | *Dracocephalum* | *ruyschiana* | Ukraine | 1969 | 50,4501 | 30,5234 | 12,6 | >1000 | S.S. Kharkevich 22.06.1969 |
| D-28 | LECB |  | *Dracocephalum* | *ruyschiana* | Ukraine | 1908 | 50,4501 | 30,5234 | 18,4 | 200 | E. Borovilovskii 21.06.1908 |
| D-29 | LECB |  | *Dracocephalum* | *ruyschiana* | Ukraine | 1965 | 49,8667 | 36,3667 | 14,3 | >1000 | N.N. Tsvelev 30.09.1965 |
| D-30 | LECB |  | *Dracocephalum* | *ruyschiana* | Ukraine | 1906 | 49,7297 | 35,63 | 17,4 | 350 | N. Androssow 07.05.1906 |
| D-128 | O |  | *Dracocephalum* | *ruyschiana* | Sweden | 1895 | na | na | 6,53 | 150 | Oscar Dalström 27.06.1895 |

**Table S2.** List of gbifIDs, used as data for ecological niche modeling, for which coordinates were lacking and estimated based on available specimen metadata (doi.org/10.15468/dl.748g3v, accessed via GBIF.org on 2021-03-13).

| **gbifID** | **Latitude** | **Longitude** | **gbifID** | **Latitude** | **Longitude** | **gbifID** | **Latitude** | **Longitude** |
| --- | --- | --- | --- | --- | --- | --- | --- | --- |
| 1096397777 | 61.1991 | 10.1443 | 1144101971 | 61.0884195 | 8.9819722 | 1144169863 | 59.8899584 | 10.5264778 |
| 1701918986 | 61.221261 | 10.100406 | 20832907 | 61.0884195 | 8.9819722 | 1144101928 | 59.8791 | 10.6149 |
| 1702400352 | 60.631038 | 10.773065 | 1702400409 | 61.066652 | 9.0230553 | 1144101957 | 59.8973 | 10.555 |
| 20832973 | 60.36727424787106 | 10.53060976965825 | 1701919012 | 61.025384 | 9.0846482 | 1144101939 | 59.8990834 | 10.577025 |
| 1144101954 | 60.81889679217599 | 11.011907769731598 | 1144101988 | 60.6298 | 10.7629 | 1701672163 | 59.8841 | 10.5353 |
| 1144101962 | 61.1565 | 10.4215 | 1701494634 | 61.2835074 | 10.3902655 | 1144101929 | 59.8899584 | 10.5264778 |
| 1702413457 | 61.1497 | 10.4258 | 1701924207 | 61.2835074 | 10.3902655 | 1144101935 | 59.8899584 | 10.5264778 |
| 1702413515 | 61.1497 | 10.4258 | 1981551005 | 61.3336 | 10.3155 | 2618440213 | 59.88753 | 10.618831 |
| 1701924243 | 61.1497 | 10.4258 | 20833005 | 61.3692 | 10.2964 | 1213527075 | 59.88753 | 10.618831 |
| 1702413439 | 61.1497 | 10.4258 | 1702413444 | 61.1437861 | 9.0693889 | 1701403425 | 59.7121611 | 10.077675 |
| 2516614697 | 61.10468 | 10.462418 | 1701494626 | 61.0690375 | 9.1665293 | 1144101951 | 59.7338 | 10.5452 |
| 1702400388 | 61.115 | 10.466111 | 1144101941 | 61.1437861 | 9.0693889 | 1702178401 | 60.8793612 | 8.4851456 |
| 1702400329 | 61.115 | 10.466111 | 1701714557 | 61.1437861 | 9.0693889 | 1701858975 | 60.1092167 | 10.2767 |
| 1699868302 | 61.115 | 10.466111 | 1702413489 | 61.0703005 | 9.1493675 | 1702400363 | 60.2397917 | 10.3870861 |
| 1096417961 | 61.03781 | 10.487958 | 1702413482 | 61.1437861 | 9.0693889 | 1701918963 | 60.2753097 | 10.4844347 |
| 1935995751 | 61.10468 | 10.462418 | 2516610664 | 59.89555771840232 | 10.7331349335398 | 1701918994 | 60.206437 | 10.3733364 |
| 1701489287 | 61.1497 | 10.4258 | 1935956276 | 59.97258 | 10.72237 | 1144101956 | 59.9408028 | 10.0015167 |
| 1702400338 | 61.115 | 10.466111 | 1095438124 | 59.97258 | 10.72237 | 1702245290 | 60.1787 | 10.3496 |
| 1702400305 | 61.115 | 10.466111 | 1710137771 | 59.898281 | 10.728876 | 1144101968 | 59.6676637 | 10.7645752 |
| 1323765186 | 61.115 | 10.466111 | 1852160033 | 59.890783 | 10.762662 | 20832977 | 60.8225 | 9.551944 |
| 1144101999 | 61.0181 | 9.2775 | 1144101919 | 59.8357 | 10.4451 | 1702413492 | 60.8225 | 9.551944 |
| 351924279 | 60.986457 | 9.131309 | 1144169842 | 59.8581986 | 10.4817274 | 1702269211 | 60.8225 | 9.551944 |
| 1702400367 | 61.6015429 | 9.7662945 | 1144101944 | 59.8484527 | 10.4731244 | 20832984 | 60.8256381 | 9.5591847 |
| 1701983033 | 60.841816 | 10.103298 | 1144101959 | 59.856476 | 10.4731357 | 1852157323 | 60.8225 | 9.551944 |
| 1701919109 | 61.529444 | 10.138889 | 1702245529 | 59.8181581 | 10.4882308 | 1701708946 | 60.8256381 | 9.5591847 |
| 1096412126 | 61.529444 | 10.138889 | 1144101932 | 59.8594 | 10.5399 | 1702400364 | 60.8198432 | 9.5634576 |
| 20832802 | 61.5268 | 10.1469 | 1144101949 | 59.8357 | 10.4451 | 1701489290 | 60.826463 | 9.547322 |
| 1702400341 | 61.5268 | 10.1469 | 1852152815 | 59.875215 | 10.527213 | 1702400307 | 60.8198432 | 9.5634576 |
| 1702413460 | 61.529444 | 10.138889 | 1852124779 | 59.875215 | 10.527213 | 1702413472 | 60.828013 | 9.52287 |
| 351924283 | 61.529444 | 10.138889 | 1144101924 | 59.8581986 | 10.4817274 | 1701714454 | 60.8225 | 9.551944 |
| 1095901699 | 60.98132 | 10.78957 | 1144101938 | 59.6060587 | 10.4441819 | 1144101966 | 60.8225 | 9.551944 |
| 20832740 | 60.92379986297257 | 10.898499644495761 | 1213527233 | 59.848078 | 10.543819 | 1144101974 | 60.8256381 | 9.5591847 |
| 1144101980 | 60.7356 | 11.2833 | 1936006303 | 59.8841 | 10.5353 | 1936625090 | 61.1696258 | 8.4267656 |
| 1701706748 | 60.7356 | 11.2833 | 2515416264 | 59.8841 | 10.5353 | 1702253866 | 61.165896 | 8.47767 |
| 1144101961 | 60.65039181637238 | 11.188820540953564 | 1936625301 | 59.88753 | 10.618831 | 1702246397 | 61.165896 | 8.47767 |
| 1701924203 | 61.0884195 | 8.9819722 | 1144101970 | 61.0884195 | 8.9819722 | 1144101978 | 61.0884195 | 8.9819722 |

**Table S3.** List of spurious occurrence records that were removed prior to ecological niche modeling, from the downloaded GBIF data (doi.org/10.15468/dl.748g3v, accessed via GBIF.org on 2021-03-13).

| **gbifID** | **gbifID** | **gbifID** | **gbifID** | **gbifID** |
| --- | --- | --- | --- | --- |
| 1702001320 | 1434673695 | 351924243 | 3045029275 | 1434675093 |
| 2398250987 | 1701919033 | 1701494555 | 3016591316 | 1434673737 |
| 1268763395 | 3043222762 | 465748884 | 3016591299 |  |
| 2409097791 | 3043096741 | 465748883 | 2516565657 |  |
| 2991868414 | 3043092748 | 465748882 | 2516536663 |  |
| 2398692682 | 1826447710 | 465748881 | 1999653264 |  |
| 1268763366 | 2251288727 | 465748880 | 1999628363 |  |
| 1268763364 | 1702319470 | 2514581061 | 1999628272 |  |
| 2516594674 | 1701924249 | 1144101950 | 1999628200 |  |
| 1702394602 | 1836424934 | 2977895802 | 1229954790 |  |
| 1323765151 | 2252366317 | 2974345407 | 121036619 |  |
| 1434676047 | 2273543612 | 3045030936 | 2411614895 |  |

**Tabell S4.**  The 95% confidence interval for the pairwise *F_ST_* estimates based on 92 SNPs from Norwegian dragonhead (NOR). The lower and upper confidence interval limit was estimated using 1000 bootstraps. The lower triangle (yellow) represents comparisons of historical regions, the upper triangle (orange) of modern regions, and the diagonal (white) comparison of the historical and modern group within similar regions. The non-overlapping 95% confidence intervals values are marked with an asterisk. Only one asterisk means that the value is lower compared to the other age group, and two asterisks means it is higher than in the other age group.

| **Regional comparison** | Oslofjorden | Tyrifjorden | Randsfjorden | Valdres | Gudbrandsdalen | Hedmark |
| --- | --- | --- | --- | --- | --- | --- |
| Oslofjorden | 0.004 - 0.008 | 0.013 - 0.034 | 0.008 - 0.017* | 0.022 - 0.039 | 0.019 - 0.037 | 0.019 - 0.035 |
| Tyrifjorden | 0.024 - 0.050 | 0.015 - 0.044 | 0.009 - 0.026* | 0.020 - 0.042 | 0.017 - 0.040 | 0.027 - 0.055 |
| Randsfjorden | 0.023 - 0.047** | 0.041 - 0.084** | 0.021 - 0.048 | 0.012 - 0.027 | 0.013 - 0.026* | 0.017 - 0.031* |
| Valdres | 0.021 - 0.038 | 0.025 - 0.051 | 0.027 - 0.060 | 0.008 - 0.018 | 0.021 - 0.045 | 0.025 -0.048 |
| Gudbrandsdalen | 0.016 - 0.039 | 0.029 - 0.061 | 0.028 - 0.061** | 0.011 - 0.025 | 0.016 - 0.035 | 0.029 - 0.056** |
| Hedmark | 0.014 - 0.033 | 0.031 - 0.072 | 0.033 - 0.070** | 0.014 - 0.030 | 0.013 - 0.026* | 0.014 - 0.031 |

**Table S5.** Measures of genetic diversity within subsampled, modern regions of Norwegian *Dracocephalum ruyschiana*. Number of individuals (N**_ind_**), alleles (N**_allele_**), polymorphic loci (N**_poly_**), observed (H**_O_**) and expected heterozygosity (H**_E_**), inbreeding coefficient (F**_IS_**), and percentage of missing data (%) was calculated for each replicated subsampling, and subsequently averaged for the separate regions. The ID of the randomly selected samples in each replicate run is provided under Sample ID.

| **Modern group** | **N_ind_** | **N_allele_** | **N_poly_** | **H_O_** | **H_E_** | **F_IS_** | **Miss (%)** | **Sample ID** |
| --- | --- | --- | --- | --- | --- | --- | --- | --- |
| Oslofjorden-run1 | 27 | 181 | 89 | 0,312 | 0,326 | 0,042 | 0,04 | 241,322,278,358,61,339,316,12,328,309,59,357,351,228,224,344,238,348,67,340,293,206,213,275,222,327,209 |
| Oslofjorden-run2 | 27 | 181 | 89 | 0,310 | 0,322 | 0,039 | 0,16 | 226,303,345,316,291,209,289,309,41,241,229,42,208,300,322,215,243,339,326,214,361,342,359,311,225,44,234 |
| Oslofjorden-run3 | 27 | 182 | 90 | 0,305 | 0,320 | 0,049 | 0,12 | 225,276,216,277,302,300,295,347,339,341,304,67,240,221,208,206,227,357,318,344,68,297,322,360,242,43,366 |
| Oslofjorden-run4 | 27 | 182 | 90 | 0,308 | 0,326 | 0,056 | 0,08 | 312,327,42,308,63,296,277,224,273,366,354,214,68,344,306,350,217,229,218,275,240,231,297,41,209,14,289 |
| Oslofjorden-run5 | 27 | 182 | 90 | 0,308 | 0,324 | 0,049 | 0,00 | 302,232,219,221,344,243,240,297,274,361,213,307,308,242,322,347,41,326,291,360,320,218,220,209,318,342,224 |
| Oslofjorden-run6 | 27 | 180 | 88 | 0,305 | 0,324 | 0,057 | 0,12 | 234,307,240,360,227,345,225,228,242,296,357,233,313,239,236,226,292,299,231,315,312,309,325,221,207,235,213 |
| Oslofjorden-run7 | 27 | 180 | 88 | 0,305 | 0,318 | 0,041 | 0,12 | 292,223,224,291,238,277,278,60,357,290,237,320,235,57,65,12,206,14,364,207,213,311,300,358,321,324,68 |
| Oslofjorden-run8 | 27 | 180 | 88 | 0,314 | 0,329 | 0,047 | 0,12 | 210,345,354,301,40,344,340,359,213,351,214,327,289,366,353,227,355,318,217,325,61,68,290,44,276,363,59 |
| Oslofjorden-run9 | 27 | 179 | 87 | 0,297 | 0,322 | 0,078 | 0,04 | 298,290,61,304,296,213,12,276,354,65,345,243,212,57,209,277,351,301,40,232,308,314,224,14,215,328,207 |
| Oslofjorden-run10 | 27 | 180 | 88 | 0,302 | 0,322 | 0,063 | 0,04 | 354,278,367,294,12,229,309,232,307,322,297,364,238,289,60,209,210,360,62,226,65,341,323,292,14,363,225 |
| **Oslofjorden-mean** | 27 | 180,70 | 88,70 | 0,306 | 0,323 | 0,052 | 0,08 |  |
| **Oslofjorden-SD** | 0 | 1,06 | 1,06 | 0,005 | 0,003 | 0,012 | 0,05 |  |
| Tyrifjorden-run1 | 5 | 162 | 70 | 0,254 | 0,315 | 0,193 | 0,22 | 24,106,112,80,167 |
| Tyrifjorden-run2 | 5 | 168 | 76 | 0,283 | 0,329 | 0,142 | 0 | 104,28,193,165,134 |
| Tyrifjorden-run3 | 5 | 169 | 77 | 0,289 | 0,315 | 0,081 | 0 | 163,334,23,79,75 |
| Tyrifjorden-run4 | 5 | 165 | 73 | 0,298 | 0,327 | 0,088 | 0,22 | 168,28,85,75,169 |
| Tyrifjorden-run5 | 5 | 161 | 69 | 0,294 | 0,302 | 0,029 | 0,22 | 92,80,170,168,109 |
| Tyrifjorden-run6 | 5 | 165 | 73 | 0,300 | 0,312 | 0,038 | 0 | 105,93,112,134,104 |
| Tyrifjorden-run7 | 5 | 163 | 71 | 0,283 | 0,314 | 0,100 | 0 | 30,76,166,332,109 |
| Tyrifjorden-run8 | 5 | 165 | 73 | 0,291 | 0,308 | 0,053 | 0 | 168,171,133,106,335 |
| Tyrifjorden-run9 | 5 | 164 | 72 | 0,267 | 0,312 | 0,143 | 0 | 283,30,338,134,109 |
| Tyrifjorden-run10 | 5 | 161 | 69 | 0,246 | 0,320 | 0,233 | 0 | 27,91,166,135,280 |
| **Tyrifjorden-mean** | 5 | 164,30 | 72,30 | 0,280 | 0,315 | 0,110 | 0,07 |  |
| **Tyrifjorden-SD** | 0 | 2,71 | 2,71 | 0,019 | 0,008 | 0,067 | 0,11 |  |
| Randsfjorden-run1 | 3 | 155 | 63 | 0,283 | 0,317 | 0,109 | 0 | 271,95,127 |
| Randsfjorden-run2 | 3 | 162 | 70 | 0,279 | 0,353 | 0,210 | 0 | 96,175,144 |
| Randsfjorden-run3 | 3 | 158 | 66 | 0,290 | 0,324 | 0,106 | 0 | 182,140,116 |
| Randsfjorden-run4 | 3 | 151 | 59 | 0,294 | 0,274 | -0,073 | 0 | 122,120,160 |
| Randsfjorden-run5 | 3 | 154 | 62 | 0,283 | 0,321 | 0,119 | 0 | 145,175,271 |
| Randsfjorden-run6 | 3 | 148 | 56 | 0,246 | 0,279 | 0,117 | 0 | 152,122,130 |
| Randsfjorden-run7 | 3 | 153 | 61 | 0,313 | 0,292 | -0,075 | 0 | 72,173,161 |
| Randsfjorden-run8 | 3 | 155 | 63 | 0,294 | 0,304 | 0,036 | 0 | 121,131,99 |
| Randsfjorden-run9 | 3 | 155 | 63 | 0,294 | 0,304 | 0,036 | 0 | 118,123,190 |
| Randsfjorden-run10 | 3 | 161 | 69 | 0,301 | 0,351 | 0,144 | 0 | 181,38,140 |
| **Randsfjorden-mean** | 3 | 155,20 | 63,20 | 0,288 | 0,312 | 0,073 | 0,00 |  |
| **Randsfjorden-SD** | 0 | 4,26 | 4,26 | 0,018 | 0,027 | 0,092 | 0,00 |  |
| Hedmark-run1 | 10 | 172 | 80 | 0,297 | 0,306 | 0,030 | 0 | 256,56,255,262,254,259,49,20,50,257 |
| Hedmark-run2 | 10 | 173 | 81 | 0,290 | 0,316 | 0,081 | 0 | 254,17,261,255,19,56,256,50,49,20 |
| Hedmark-run3 | 10 | 167 | 75 | 0,263 | 0,287 | 0,082 | 0 | 262,260,263,17,256,56,257,261,18,50 |
| Hedmark-run4 | 10 | 170 | 78 | 0,270 | 0,295 | 0,088 | 0 | 19,259,256,263,254,20,262,255,17,261 |
| Hedmark-run5 | 10 | 170 | 78 | 0,271 | 0,301 | 0,101 | 0 | 17,19,260,255,261,20,18,258,259,49 |
| Hedmark-run6 | 10 | 171 | 79 | 0,279 | 0,293 | 0,047 | 0 | 257,49,258,17,18,19,262,260,263,20 |
| Hedmark-run7 | 10 | 173 | 81 | 0,276 | 0,304 | 0,092 | 0 | 56,263,257,258,50,259,256,49,261,254 |
| Hedmark-run8 | 10 | 169 | 77 | 0,253 | 0,289 | 0,122 | 0 | 49,261,260,17,263,20,254,255,56,257 |
| Hedmark-run9 | 10 | 174 | 82 | 0,280 | 0,300 | 0,065 | 0 | 261,260,259,258,256,254,262,50,49,20 |
| Hedmark-run10 | 10 | 169 | 77 | 0,248 | 0,287 | 0,138 | 0 | 49,19,261,255,263,262,17,254,50,260 |
| **Hedmark-mean** | 10 | 170,80 | 78,80 | 0,273 | 0,298 | 0,085 | 0,00 |  |
| **Hedmark-SD** | 0 | 2,20 | 2,20 | 0,015 | 0,009 | 0,032 | 0,00 |  |
